# Obesity‐Associated TRIM15 Promotes the Proliferation of Esophageal Adenocarcinoma Through the YY2/FOXRED1 Axis

**DOI:** 10.1002/advs.202417330

**Published:** 2025-11-14

**Authors:** Haohui Wang, Chong Yang, Dayuan Luo, Pingting Liu, Zhen Zeng, Weilin Peng, Dongzi Peng, Hao Su, Xiaoxiong Xiao, Haiqin Wang, Xin Jin

**Affiliations:** ^1^ Department of Thoracic Surgery The Second Xiangya Hospital Central South University Changsha Hunan 410011 China; ^2^ Hunan Key Laboratory of Early Diagnosis and Precise Treatment of Lung Cancer The Second Xiangya Hospital Central South University Changsha Hunan 410011 China; ^3^ Department of Hepatobiliary and Pancreatic Surgery Sichuan Provincial People's Hospital University of Electronic Science and Technology of China Chengdu 610072 China; ^4^ Department of Health Management Center Hunan Provincial Maternal and Child Health Care Hospital Changsha Hunan 410008 China; ^5^ Department of Geriatrics The Second Xiangya Hospital Central South University Changsha Hunan 410011 China; ^6^ Hunan Clinical Medical Research Center for Geriatric Syndrome Changsha Hunan 410011 China; ^7^ Department of Gastroenterology The Second Xiangya Hospital Central South University Changsha Hunan 410011 China; ^8^ Research Center of Digestive Disease Central South University Changsha Hunan 410011 China; ^9^ Department of Urology The Second Xiangya Hospital Central South University Changsha Hunan 410011 China; ^10^ Uro‐Oncology Institute of Central South University Changsha Hunan 410011 China; ^11^ Furong Laboratory Central South University Changsha Hunan 410000 China; ^12^ Department of Thoracic Surgery Xiangya Hospital Central South University Changsha Hunan 410008 China; ^13^ Xiangya Lung Cancer Center Xiangya Hospital Central South University Changsha Hunan 410008 China

**Keywords:** TRIM15, esophageal adenocarcinoma, ferroptosis, lipid metabolism, obesity

## Abstract

Obesity has been identified as an independent risk factor for gastroesophageal reflux disease (GERD) and esophageal adenocarcinoma (EAC). Oxidative stress and inflammation driven by chronic GERD are the main causes of the tumorigenesis of EAC, but the underlying mechanism remains elusive. Here, the inflammation‐upregulated E3 ligase, tripartite motif 15 (TRIM15), is identified as a key driver of obesity‐associated EAC. TRIM15 promotes the degradation of YY2 is demonstrated through the ubiquitin‐proteasome system, which in turn dysregulates lipid metabolism and enhances the proliferation of EAC cells. Furthermore, YY2 transcriptionally is shown that increases FOXRED1 expression. FOXRED1 is subsequently identified as an essential effector for the TRIM15‐induced dysregulation of lipid and energy metabolism in EAC cells. Thus, a novel obesity‐associated TRIM15/YY2/FOXRED1 axis is identified that contributes to the proliferation of EAC. Given that lipid metabolism regulates ferroptosis by controlling cellular processes associated with phospholipid peroxidation. The TRIM15/YY2/FOXRED1 axis demonstrates that it modulates SLC3A2 expression via the mTOR/c‐MYC pathway, thereby regulating GPX4 levels to influence EAC sensitivity to ferroptosis‐inducing compounds and proposing a therapeutic strategy for EAC.

## Graphical abstract

The study identifies TRIM15 as a key driver in the development of obesity‐associated esophageal adenocarcinoma (EAC). Mechanistically, TRIM15 degrades YY2 through the proteasome pathway, suppressing *FOXRED1* transcription and ultimately accelerating tumor proliferation. We further characterize the critical role of the TRIM15/YY2/FOXRED1 axis in the dysregulation of lipid metabolism and ferroptosis in EAC.

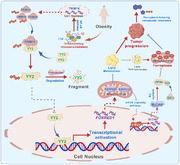

## 1 Introduction

Obesity has been identified as an independent risk factor for gastroesophageal reflux disease (GERD) and esophageal adenocarcinoma (EAC).^[^ ^1^, ^2^ ^]^ The relative risk of EAC in obese individuals with a high body mass index (BMI) is 4.8 times higher than in those with a normal BMI.^[^ ^3^ ^]^ Oxidative stress and inflammation driven by chronic GERD are the main causes of EAC carcinogenesis.^[^ ^2^ ^]^ Several studies have shown that stimulation by gastric acid, bile acids, and inflammatory factors can induce excessive activation of the NF‐κB pathway in the columnar epithelial cells of the esophageal mucosa, leading to the differentiation of the columnar epithelium into intestinal epithelial cells, ultimately inducing the cancerous transformation of Barrett's esophagus (BE) to EAC.^[^ ^4^, ^5^ ^]^ Despite extensive research, the precise pathophysiology of EAC remains unclear. Further investigation is required to elucidate the role of hormonal and molecular oncogenic pathways in the aetiology of EAC in obese patients.

Obesity‐associated metabolic dysregulation often manifests in cancer through aberrant activation of lipid biosynthesis.^[^ ^1^ ^]^ Dysregulation of lipid metabolism plays a pivotal role in the metabolic adaptation of tumors and contributes to treatment resistance, metastatic dissemination, and secondary tumor growth.^[^ ^6^, ^7^ ^]^ Levels of enzymes responsible for lipid elongation, Stearoyl‐CoA Desaturases (SCD) and fatty acid desaturases (FADS), are elevated in various obesity‐associated cancers.^[^ ^8^, ^9^ ^]^ A comprehensive understanding of how obesity‐associated abnormalities in lipid metabolism affect tumor progression is essential for developing novel cancer therapies.

Consistent with previous findings, we conducted a preliminary meta‐analysis demonstrating that obesity is a key risk factor for EAC. Subsequently, to elucidate the changes of obesity‐associated metabolic dysregulation during EAC progression, we established a subcutaneous EAC xenograft model in nude mice after feeding with a high‐fat diet (HFD) or a normal diet (ND). The results showed that HFD induced EAC cell proliferation and activated multiple oncogenic pathways. Notably, E3 ubiquitin‐protein ligase TRIM15 was significantly upregulated in tumors associated with an HFD and in EAC tissues derived from patient samples. We previously reported that TRIM15 dysregulated lipid metabolism to promote pancreatic cancer progression.^[^ ^10^ ^]^ We also found that TRIM15 activated the AKT signaling pathway to decrease the sensitivity of liver cancer cells to tyrosine kinase inhibitors.^[^ ^11^ ^]^ Meanwhile, TRIM15 is considered an inflammation‐related protein regulated by TNF‐α, and it inhibits TLR4.^[^ ^12^, ^13^ ^]^ In this study, we demonstrated that TRIM15 is a key molecule in the obesity‐induced progression of EAC by degrading YY2, which in turn inhibits *FOXRED1* transcription to regulate lipid and energy metabolism. Given that mitochondrial structural and functional abnormalities are closely associated with ferroptosis,^[^ ^14^ ^]^ and lipid metabolism regulates ferroptosis by controlling cellular processes associated with phospholipid peroxidation.^[^ ^15^, ^16^ ^]^ Our study demonstrates that the TRIM15/YY2/FOXRED1 axis modulates the sensitivity of EAC cells to the ferroptosis‐inducing compounds such as RSL3 and imidazole ketone erastin (IKE), providing a strategy for the clinical treatment of EAC.

## 2 Results

### 2.1 TRIM15 Promotes the Proliferation of Obesity‐Associated EAC

**Figure 1:**
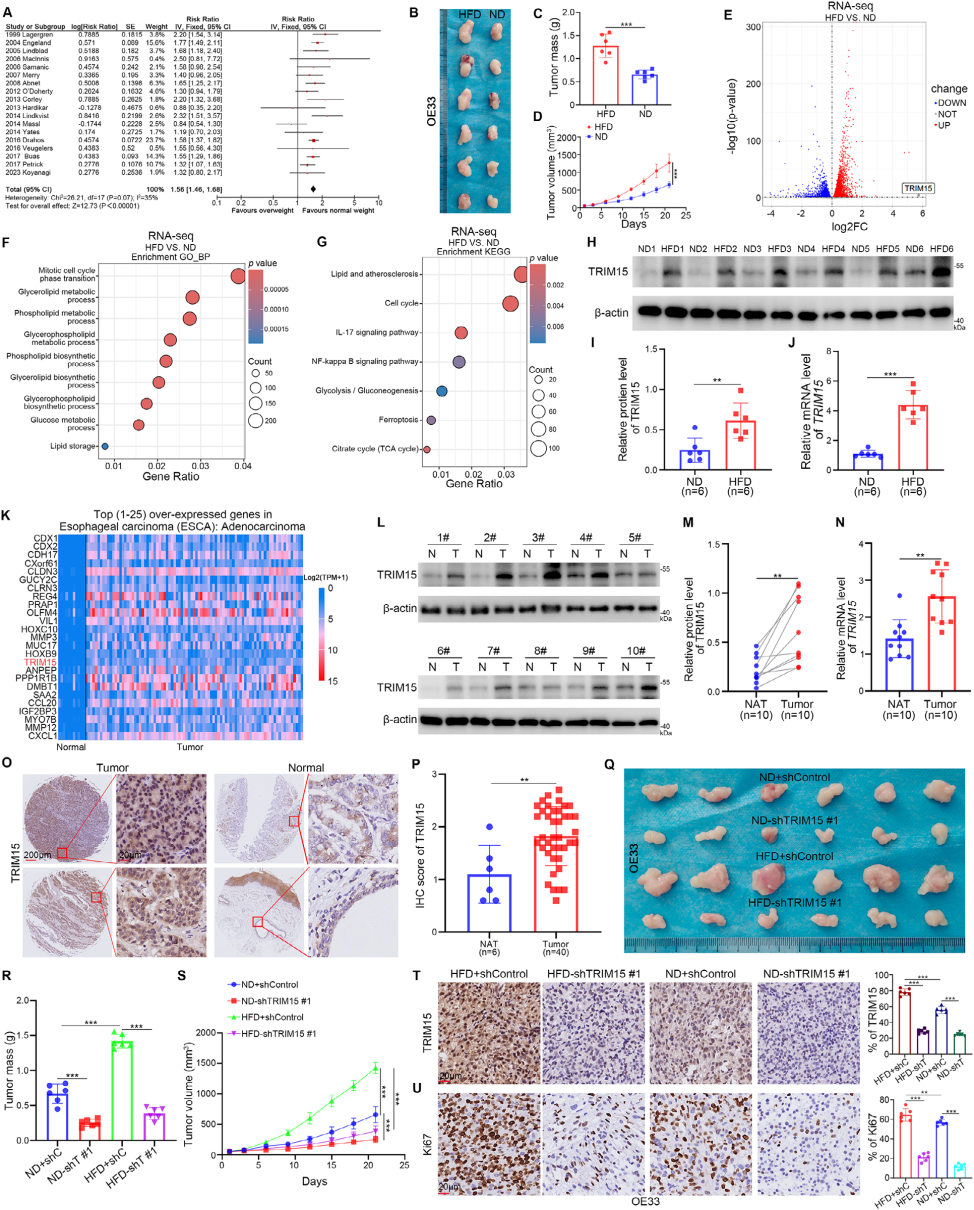
TRIM15 is a key molecule in obesity‐associated EAC. A) Meta‐analysis of the effect of BMI on EAC. B–D) Nude mice were divided into HFD and ND groups and fed for 8 weeks, OE33 cells were subcutaneously injected into the nude mice. Tumor image (B), tumor mass (C), tumor growth curve (D). Data presented as mean ± SEM with six replicates. E–G) The transcriptome sequencing analysis of the transplanted tumors in HFD and ND groups. Volcano map (E), GO‐BP (F), and KEGG (G) enrichment analyses of the RNA‐seq data. *p* values as indicated. H–J) The protein and mRNA levels of TRIM15 from the transplanted tumors in HFD and ND groups were examined by Western blotting (H) and RT‐qPCR (J), and the protein level of TRIM15 was quantified by ImageJ software (I). Data presented as mean ± SEM with three replicates. ^**^ *p* < 0.01; ^***^ *p* < 0.001. K) The UALCAN‐TCGA data platform analysis revealed the top 25 highly expressed genes in EAC tumor tissues compared to normal esophageal tissues. L–N) The protein and mRNA levels of TRIM15 from patients with EAC were examined by Western blotting (L) and RT‐qPCR (N), and the protein level of TRIM15 was quantified by ImageJ software (M). Data presented as mean ± SEM with three replicates. ^**^ *p* < 0.01. O and P) The EAC tissue microarray was analyzed by IHC staining by using the anti‐TRIM15 antibody. ^**^ *p* < 0.01. Q–U) OE33 cells were transfected with shTRIM15 plasmids for 72 h. After puromycin selection, these cells were subcutaneously injected into the nude mice. These mice were fed for HFD or ND. Tumor image (Q), tumor mass (R), tumor growth curve (S), IHC staining by using the anti‐TRIM15 (T) or anti‐Ki67 antibody (U). Data presented as mean ± SEM with six replicates. ^**^ *p* < 0.01; ^***^ *p* < 0.001.

To investigate the potential association between obesity and EAC, we conducted a meta‐analysis demonstrating that obesity acts as a risk factor for developing EAC and is closely associated with EAC progression (HR=1.56, 95% CI: 1.46‐1.68, *p* < 0.00001, I^2^=35%) (**Figure** **1**A). To investigate the effect of an obesity‐induced oncogenic microenvironment on EAC cell proliferation, nude mice were randomized into two groups receiving an HFD or an ND. After 8 weeks of feeding, EAC cells were subcutaneously injected into the mice. Following an additional 3 weeks, xenografts were excised and subjected to RNA‐seq analysis (Figure S1A, Supporting Information). Mice in the HFD group weighed significantly more than those in the ND group (Figure S1B, Supporting Information) and had significantly larger subcutaneous tumor mass and volume (Figure 1B–D). The Gene Ontology‐Biological Process (GO‐BP) (Figure 1F) and Kyoto Encyclopedia of Genes and Genomes (KEGG) (Figure 1G) enrichment analyses of the RNA‐seq data of the xenografts showed that the lipid metabolism and energy metabolism pathways were significantly dysregulated in the HFD group compared to the ND group. Notably, *TRIM15* exhibited the highest fold change in the HFD group compared to the ND group (Figure 1E; Figure S1H, Supporting Information). Western blotting and RT‐qPCR assays revealed a significant upregulation of TRIM15 expression in EAC graft tumors of the HFD group compared to those from the ND group (Figure 1H–J). Interestingly, the UALCAN‐TCGA data platform analysis (https://ualcan.path.uab.edu/analysis.html) revealed that *TRIM15* was among the top 25 most highly expressed genes in EAC tumor tissues compared to normal esophageal tissues (Figure 1K). Several studies have shown that the TRIM family is involved in regulating the progression of multiple obesity‐associated diseases.^[^ ^17^, ^18^, ^19^ ^]^ However, the role of the TRIM family in obesity‐associated EAC progression is unclear. Since we previously reported that TRIM15 dysregulated lipid metabolism to promote pancreatic cancer progression,^[^ ^10^ ^]^ and is considered an inflammation‐related protein regulated by TNF‐alpha and inhibits TLR4.^[^ ^12^, ^13^ ^]^ We decided to study the role of TRIM15 in obesity‐associated EAC.

To explore the effects of HFD on endocrine metabolic levels in mice, we assessed changes in fasting glucose, lipids, and inflammatory indexes in the blood of mice. We observed a significant increase in TG, CHO, LDL, HDL, TNF‐α, IL‐6, IL‐1β, and HOMA‐IR in the HFD group of mice compared to the ND group (Figure S1C–E, Supporting Information). Subsequently, we found that the addition of HFD group mouse plasma to EAC cells significantly promoted cell proliferation (Figure S1F,G, Supporting Information). Interestingly, we found that the plasma of mice in the HFD group significantly elevated the protein and mRNA levels of TRIM15 in EAC cells (Figure S1I,J, Supporting Information). In addition, the UALCAN‐TCGA data platform analysis revealed that the overall survival time was shorter in EAC patients with high *TRIM15* expression (Figure S2A, Supporting Information), and the expression of *TRIM15* in esophageal cancer tumor tissues was elevated with the increasing BMI of patients (Figure S2B, Supporting Information). Western Blotting and RT‐qPCR analyses conducted on clinical tissue specimens, as well as immunohistochemistry (IHC) experiments performed on tissue microarray specimens (No.D046Ca01, Bioaitech, China), consistently demonstrated that the expression of TRIM15 was elevated in EAC tumor tissues (Figure 1L–P). EAC originates from glandular cells with dysplasia in the lower esophagus.^[^ ^20^ ^]^ To clarify whether TRIM15 is upregulated during the neoplastic progression of BE. Interestingly, we found that TRIM15 stained more deeply in glandular cells with dysplasia (disorganized cell arrangement, markedly enlarged nuclei, irregular nuclear membranes, and loss of nuclear polarity) than those without dysplasia (neater cell arrangement, no markedly enlarged nuclei, regular nuclear membranes, and absence of nuclear lamina propria) in BE (Figure S2C, Supporting Information). Moreover, the top “Normal” TMA specimen favors BE tissue, while the lower “Normal” TMA specimen favors normal esophageal tissue (Figure 1O). Notably, we found that TRIM15 staining in the glandular cells of BE tissue was stronger than that of the glandular cells in normal esophageal tissue, whereas TRIM15 was more strongly expressed in EAC tissues (Figure 1O). Our results suggest that TRIM15 may be involved in the neoplastic process from BE to EAC.

Thus, it is reasonable to hypothesize that TRIM15 plays a key role in obesity‐associated EAC progression. To determine the effect of TRIM15 on EAC cells in vivo, TRIM15 knockdown cell lines were generated using TRIM15‐specific shRNAs prior to subcutaneous tumorigenesis in nude mice. The results showed that the mass and volume of EAC tumors were significantly reduced after TRIM15 knockdown in both the HFD and ND groups (Figure 1Q–S; Figure S2D–F, Supporting Information). IHC analysis revealed that TRIM15 knockdown significantly inhibited the expression of Ki67, a cell proliferation marker, in both the HFD and ND groups (Figure 1T,U). Analysis of RNA‐seq data from tumors revealed that transcriptional levels of *IL‐1α*, *IL‐1β*, *IL‐6*, and numerous other NF‐κB^[^ ^21^ ^]^ pathway‐related inflammatory factors were upregulated in the HFD group (Figure S1K, Supporting Information). TNF‐α and IL‐6, as important pro‐inflammatory factors, are involved in a variety of pathological mechanisms, including tumors.^[^ ^21^, ^22^ ^]^ We found that TNF‐α, IL‐6 were elevated in the plasma of mice in the HFD group compared to the ND group (Figure S1E, Supporting Information). Meanwhile, previous studies have shown that TNF‐α induces up‐regulation of TRIM15 expression in breast cancer cells through activation of the NF‐κB pathway.^[^ ^12^ ^]^ Similarly, the KEGG enrichment analyses of the RNA‐seq data of the HFD and the ND groups’ xenografts showed that the NF‐κB pathway was significantly dysregulated (Figure 1G). Then, we noticed that TNF‐α and IL‐6 could increase the phosphorylation of p65 and TRIM15 expression in EAC cells (Figure S1L,M, Supporting Information), and the effects of TNF‐α/IL‐6‐induced up‐regulation of TRIM15 were decreased in NF‐κB inhibitor treatment group (Figure S1N, Supporting Information). Moreover, the ability of TNF‐α or IL‐6 to promote TRIM15 promoter activity was blocked following the addition of a specific NF‐κB inhibitor (Figure S1O, Supporting Information). Together, these results suggest that high inflammation levels lead to a significant upregulation of TRIM15 in obesity‐associated EAC tumor tissues, and knockdown of TRIM15 in EAC cells significantly inhibits the proliferative effects of obesity on EAC.

### 2.2 TRIM15 Promotes EAC Proliferation and Dysregulates Lipid Metabolism

**Figure 2:**
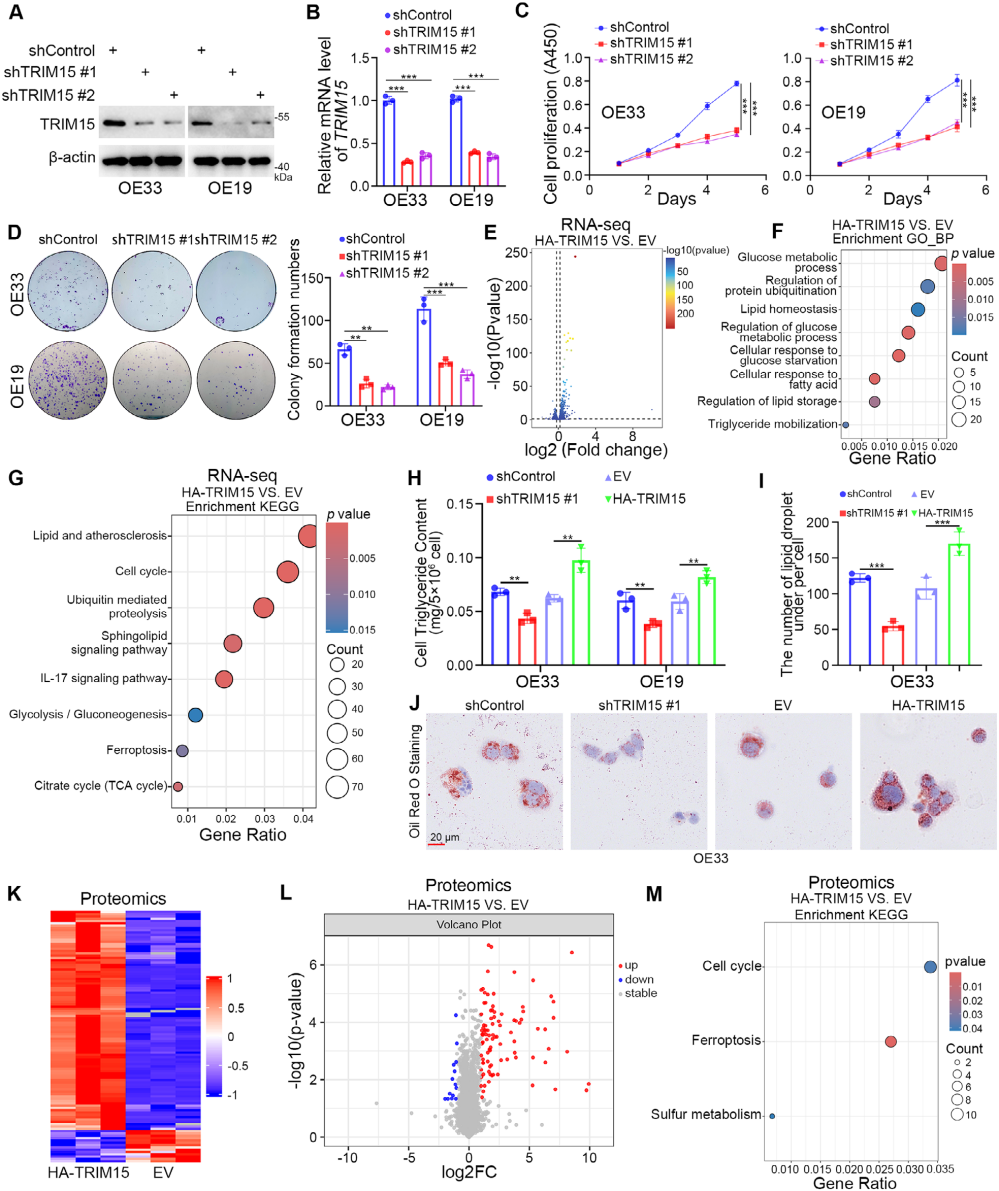
TRIM15 promotes EAC progression and dysregulates lipid metabolism. A–D) EAC cells were transfected with shTRIM15 plasmids for 72 h. Cells were collected for Western blot analysis (A), RT‐qPCR analysis (B), CCK‐8 assay (C), and colony formation assay (D). Data presented as mean ± SEM with three replicates. ^**^ *p* < 0.01; ^***^ *p* < 0.001. E–G) The transcriptome sequencing analysis of OE33 cells after transfection with EV or HA‐TRIM15 for 48 h. Volcano map (E), GO‐BP (F), and KEGG enrichment analyses (G) of the RNA‐seq data after overexpression of TRIM15 in OE33 cells. *p* values as indicated. H–J) EAC cells were transfected with the indicated constructs for 48 or 72 h. Cellular triglyceride assay (H), Oil Red O Staining (I and J). Data presented as mean ± SEM with three replicates. ^**^ *p* < 0.01; ^***^ *p* < 0.001. K–M) The proteomics analysis of OE33 cells after transfection with EV or HA‐TRIM15 for 48 h. Heat map (K), volcanic map (L), and KEGG enrichment analysis (M) of the proteomics data after overexpression of TRIM15 in OE33 cells. *p* values as indicated.

We next investigated the cancer‐related function of TRIM15 in EAC cells. Knockdown of TRIM15 significantly inhibited cell proliferation (**Figure** **2**A–D), while overexpression of TRIM15 produced the opposite results (Figure S3A–D, Supporting Information). Next, we overexpressed TRIM15 in OE33 cells and conducted RNA‐seq analysis (Figure 2E). The GO‐BP (Figure 2F) and KEGG (Figure 2G) enrichment analyses indicated that overexpression of TRIM15 significantly dysregulated lipid and energy metabolism‐related pathways, including the Cellular response to glucose starvation, the Cellular response to fatty acids, the Regulation of glucose metabolic process, the Glucose metabolic process, the Regulation of lipid storage, the Triglyceride mobilization, the Lipid homeostasis, the Lipid and atherosclerosis, the IL‐17 signaling pathway, the Sphingolipid signaling pathway, the Citrate cycle (TCA cycle), and the Glycolysis/Gluconeogenesis.

Our previous study showed that TRIM15 promotes pancreatic cancer progression by dysregulating lipid metabolism.^[^ ^10^ ^]^ Combined with the RNA‐seq analysis of TRIM15 in EAC, we speculated that TRIM15 promotes the progression of EAC by disrupting lipid metabolism. Similarly, knockdown of TRIM15 significantly reduced triglyceride (TG) and lipid droplet levels in EAC cells, whereas overexpression of TRIM15 yielded the opposite result (Figure 2H–J). Subsequently, to identify the downstream substrates of TRIM15, we overexpressed TRIM15 in OE33 cells and conducted proteomic analysis. The heat map (Figure 2K) and volcano map (Figure 2L) showed that overexpression of TRIM15 in EAC cells results in a large number of altered protein levels. The Venn diagram in the proteomic showed that out of 6784 proteins exhibiting differences, 26 proteins were detected only in the TRIM15 overexpression group and 27 proteins were detected only in the EV group (Figure S3E, Supporting Information). The KEGG enrichment analysis of the proteomics results revealed that overexpression of TRIM15 was significantly associated with the Cell cycle, the Ferroptosis, and the Sulfur metabolism pathway (Figure 2M). The Domain (Figure S3F, Supporting Information) and GO (Figure S3G, Supporting Information) enrichment analyses showed that the structure and function of multiple molecules are affected, with a major concentration and interaction in the nucleus (Figure S3H,I, Supporting Information). Together, these results suggest that TRIM15 promotes EAC cells' proliferation by dysregulating lipid metabolism. Further investigation is required to elucidate the underlying mechanisms.

### 2.3 TRIM15 Promotes YY2 Degradation Through the K48‐Linked Ubiquitin‐Proteasome System

**Figure 3:**
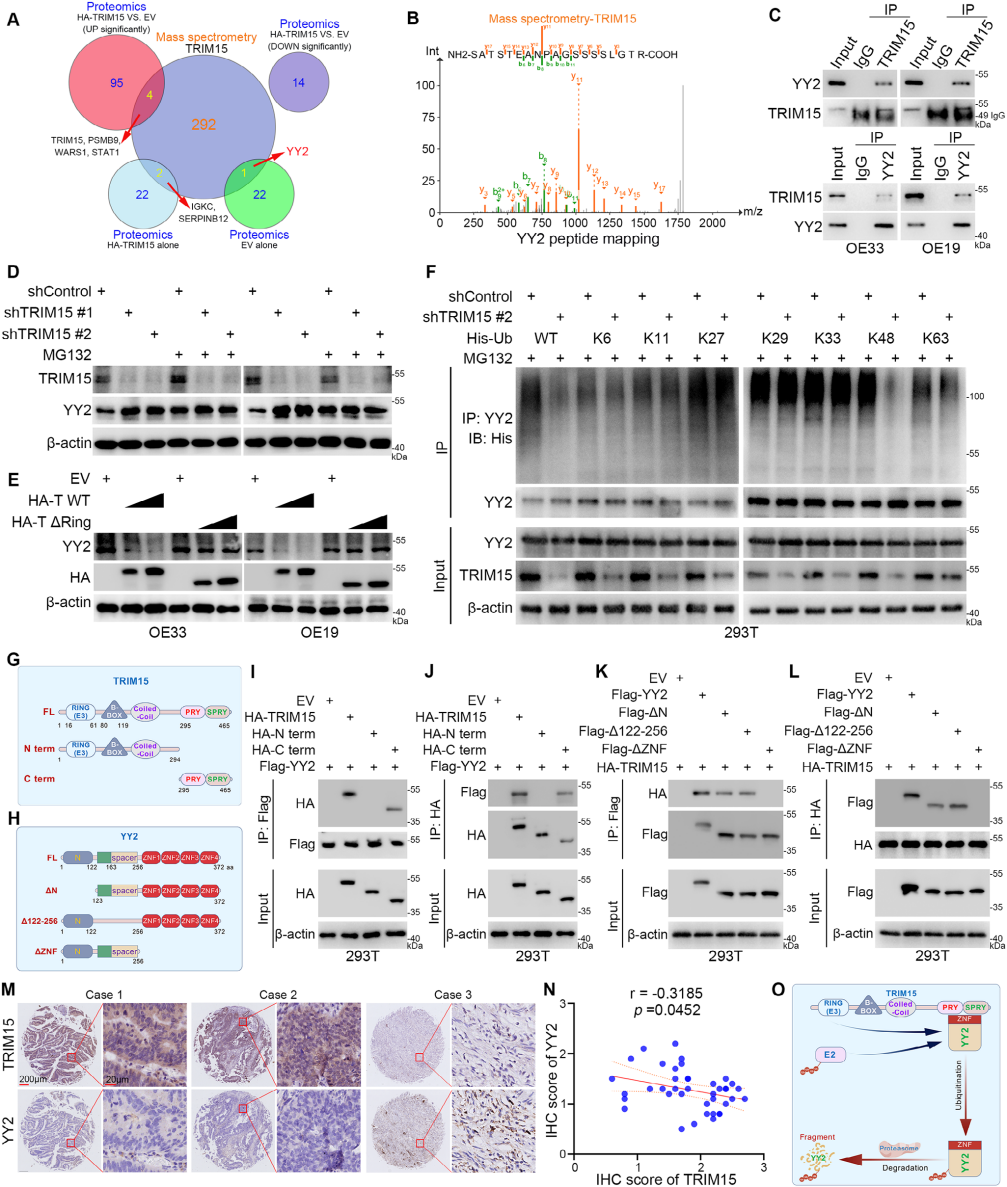
TRIM15 promotes YY2 degradation through the ubiquitin proteasome system. A) A Venn diagram of TRIM15 mass spectrometry analysis and proteomics takes an intersection. B) Binding sites of TRIM15 and YY2 in mass spectrometry analysis. C) EAC cells were infected with Flag‐YY2 plasmids. After 48 h, Co‐IP was performed, and the IP samples were analyzed through Western blot analysis. D) EAC cells were transfected with shTRIM15 plasmids for 48 h, cells were treated with MG132 (10 µM) for another 24 h. Then cells were collected for Western blot analysis. E) EAC cells were transfected with HA‐TRIM15 and HA‐∆RING plasmids for 48 h, and cells were collected for Western blot analysis. F) 293T cells were infected with shTRIM15 and His‐Ub plasmids for 48 h, cells were treated with MG132 (10 µM) for another 24 h. Then cells were collected for Western blot analysis. G and H) Diagrammatic presentation of TRIM15 (G) and YY2 (H) protein full length and various deletion mutants. I–L) 293T cells were infected with the corresponding plasmids. After 48 h, Co‐IP was performed, and the IP samples were analyzed through Western blot analysis. M and N) The EAC tissue microarray was analyzed by IHC staining by using the anti‐TRIM15 or anti‐YY2 antibody. *p* values as indicated. O) The model diagram of TRIM15 regulating YY2 protein changes by the ubiquitination pathway to promote EAC progression.

Studies have demonstrated that TRIM15 frequently functions as an E3 ubiquitin ligase, facilitating the ubiquitination of substrate proteins.^[^ ^23^ ^]^ Subsequently, we performed mass spectrometry analysis against TRIM15 in OE33 cells. Combining the results of significant differences and presence/absence in TRIM15 proteomics and intersecting with the results of mass spectrometry analysis, seven proteins (YY2, IGKC, SERPINB12, TRIM15, PSMB9, WARS1, and STAT1) were identified (**Figure** **3**A). Interestingly, by analyzing the proteomic results of TRIM15, we found that only YY2 was present in the EV group. Therefore, we focused on the YY2 protein. The proteomics and mass spectrometry analyses in EAC cells indicate that TRIM15 may bind to YY2 (Figure 3A,B). The mutual binding of TRIM15 to YY2 was further confirmed by immunoprecipitation (IP) experiments in EAC and 293T cells (Figure 3C; Figure S4A, Supporting Information). Similarly, the glutathione S‐transferase (GST) pull‐down experiment demonstrated that TRIM15 binds to YY2 in vitro (Figure S4J, Supporting Information). We then showed that knockdown of TRIM15 in EAC cells increased the protein level of YY2 (Figure S4B, Supporting Information), but did not affect the mRNA level of *YY2* (Figure S4C, Supporting Information). These findings suggest that TRIM15 may regulate the protein stability of YY2.

Subsequently, EAC cells were treated with the 26S proteasome inhibitor MG132. The results showed that MG132 treatment diminished the effect of TRIM15 on YY2 protein levels, and the addition of the autophagy inhibitor chloroquine (CQ) did not attenuate the reduction of YY2 protein by overexpression of TRIM15 in EAC or 293T cells (Figure 3D; Figure S4D,E, Supporting Information). Furthermore, the TRIM15‐∆Ring plasmid was designed to eliminate the E3 ligase function of ubiquitination modification of TRIM15.^[^ ^11^ ^]^ We found that TRIM15‐∆Ring no longer downregulated the protein level of YY2 (Figure 3E). Concurrently, knockdown of TRIM15 prolonged the protein half‐life of YY2 (Figure S4F,G, Supporting Information), while overexpression of TRIM15 shortened the protein half‐life of YY2 (Figure S4H,I, Supporting Information). However, the TRIM15‐∆Ring did not shorten the protein half‐life of YY2 in EAC cells (Figure S4H,I, Supporting Information). In addition, we observed that knockdown of TRIM15 in 293T cells resulted in decreased levels of polyubiquitination of YY2 (Figure S4K, Supporting Information), whereas overexpression of TRIM15 resulted in increased levels of polyubiquitination of YY2 (Figure S4L, Supporting Information). Similarly, TRIM15‐ΔRing did not elevate the polyubiquitination level of YY2 (Figure S4L, Supporting Information). Then, we found that knockdown of TRIM15 decreased the total ubiquitination and K48‐linked ubiquitination of YY2 in 293T cells (Figure 3F). In contrast, TRIM15 overexpression enhanced K48‐linked ubiquitination of YY2 in 293T cells (Figure S4M, Supporting Information). Mapping the TRIM15 and YY2 regions according to their functional features, two truncated mutants (N term and C term) were constructed for TRIM15 (Figure 3G) according to our previous study,^[^ ^10^ ^]^ and three truncated mutants (ΔN, Δ122‐256, and ΔZNF) were constructed for YY2 (Figure 3H). The Co‐IP assays showed that the C‐terminal region of TRIM15 mediated the physical interactions with YY2 (Figure 3I,J). Similarly, the ZNF region of YY2 is critical for interactions with TRIM15 (Figure 3K,L). Furthermore, the IHC results in EAC tissue microarrays demonstrated a negative correlation between TRIM15 and YY2 expression levels (Spearman's correlation coefficient *r* = ‐0.3185, *p* = 0.0452) (Figure 3M,N). Thus, these results suggest that the stability of YY2 protein in EAC cells is regulated through the K48‐linked ubiquitination process mediated by TRIM15 (Figure 3O).

### 2.4 YY2 Acts as the Mediator for TRIM15 in EAC Cells to Regulate Lipid Metabolism

**Figure 4:**
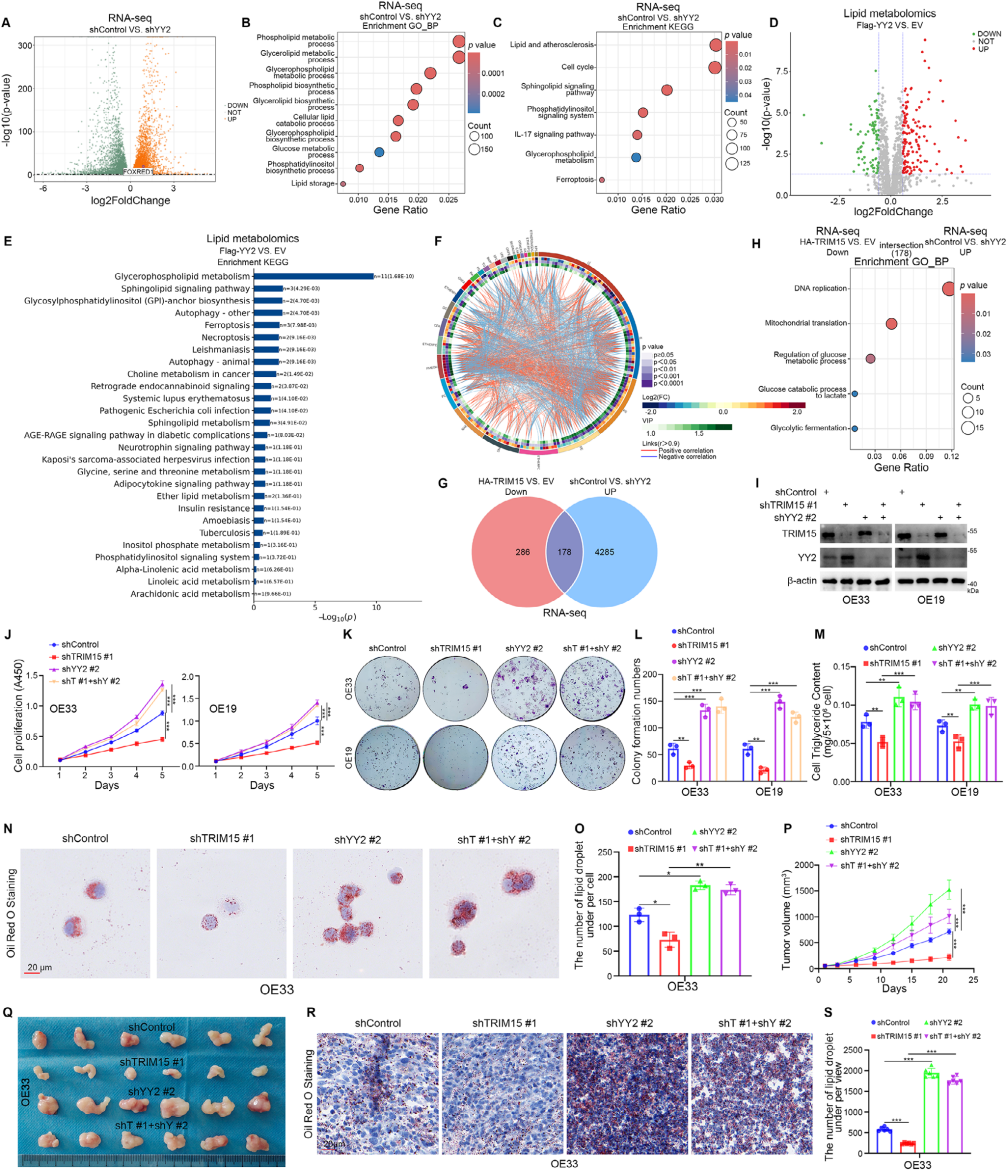
YY2 acts as the mediator for TRIM15 in EAC cells. A–C) The transcriptome sequencing analysis of OE33 cells after transfection with shYY2 or shControl for 72 h. Volcano map (A), GO‐BP (B), and KEGG enrichment analyses (C) of the RNA‐seq data after knockdown of YY2 in OE33 cells. *p* values as indicated. D–F) The lipid metabolomics analysis of OE33 cells after transfection with EV or Flag‐YY2 plasmids for 48 h. Volcano map (D), KEGG enrichment analysis (E), Circos chord plot (From the outside to the inside are the names of the lipid molecules, classification, multiplicity of differences, *p*‐value, OPLS‐DA VIP, and correlation linkage (positive correlation in red, negative correlation in blue)) (F). *p* values as indicated. G) Venn diagram of intersecting rows of RNA‐seq results for TRIM15 and YY2. H) KEGG enrichment analysis of intersecting rows of RNA‐seq results for TRIM15 and YY2. *p* values as indicated. I–O) EAC cells were transfected with shTRIM15 or shYY2 plasmids for 48 h. Cells were collected for Western blot analysis (I), CCK‐8 assay (J), colony formation assay (K and L), cellular triglyceride assay (M), and Oil Red O Staining (N and O). Data presented as mean ± SEM with three replicates. ^*^ *p* < 0.05; ^**^ *p* < 0.01; ^***^ *p* < 0.001. P–S) OE33 cells were transfected with shTRIM15 or/and shYY2 plasmids for 72 h. After puromycin selection, these cells were subcutaneously injected into the nude mice. Tumor image (Q), tumor growth curve (P), and Oil Red O Staining (R and S). Data presented as mean ± SEM with six replicates. ^***^ *p* < 0.001.

Previous studies have shown that YY2 is involved in tumor progression as a tumor suppressor gene.^[^ ^24^, ^25^ ^]^ Not surprisingly, we observed that knockdown of YY2 in EAC cells significantly enhanced cell proliferation (Figure S5A–D, Supporting Information), while overexpression of YY2 yielded the opposite results (Figure S5E–H, Supporting Information). Subsequently, we performed RNA‐seq analysis after knocking down YY2 in OE33 cells (**Figure** **4**A). Interestingly, the GO‐BP (Figure 4B) and KEGG (Figure 4C) enrichment analyses showed that knockdown of YY2 regulated numerous pathways associated with lipid and glucose metabolism. Thus, we then overexpressed YY2 in OE33 cells for lipid metabolomics analysis. The Principal Component Analysis (PCA) and Partial Least Squares Discriminant Analysis (PLS‐DA) of lipid metabolomics analyses reported for YY2 indicated significant metabolite differences between the two groups, and the results were stable and reliable (Figure S6A–D, Supporting Information). The heatmap (Figure S6E, Supporting Information) and volcano plots (Figure 4D) indicate that multiple lipid abundances were affected. The results of the Circos chord plot of YY2 lipid metabolomics showed significant associations between different lipids (Figure 4F). A wide range of lipids, including cardiolipins (CLs), phosphatidylglycerols (PGs), phosphatidylcholines (PCs), phosphatidylethanolamines (PEs), and TG, were highly altered (Figure S6F–J, Supporting Information). The KEGG enrichment analysis demonstrated that YY2‐regulated lipid metabolites were significantly correlated with the Glycerophospholipid metabolism, the Sphingolipid signaling pathway, the Glycosylphosphatidylinositol (GPI)‐anchor biosynthesis, the Autophagy‐other, and the Ferroptosis (Figure 4E). We also showed that knockdown of YY2 resulted in increased levels of TGs and lipid droplets, whereas overexpression of YY2 decreased the levels of TGs and lipid droplets in EAC cells (Figure S5I,J, Supporting Information).

In addition, 178 genes were found to be co‐regulated by intersecting the RNA‐seq results of TRIM15 and YY2 (Figure 4G). The GO‐BP enrichment analysis showed that the co‐regulated genes were significantly correlated with the DNA replication, the Regulation of glucose metabolic process, the Glucose catabolic process to lactate, and the Glycolytic fermentation (Figure 4H). Furthermore, we observed that simultaneous knockdown of TRIM15 and YY2 in EAC cells counteracted the inhibitory effect of TRIM15 knockdown alone on cell proliferation (Figure 4I–L; Figure S5K–M, Supporting Information). Additionally, the decreasing effect of single knockdown of TRIM15 on TG and lipid droplet levels in EAC cells was counteracted by simultaneous knockdown of TRIM15 and YY2 (Figure 4M–O). Animal studies showed that simultaneous knockdown of TRIM15 and YY2 counteracted the inhibitory effect of TRIM15 knockdown alone on the proliferative capacity of EAC cells (Figure 4P,Q), and the results of tumor oil red O staining indicated that the inhibition of lipid droplet levels was also counteracted (Figure 4R,S). Together, these results suggest that YY2 might be a mediator for TRIM15‐induced EAC cell proliferation and lipid metabolism dysregulation.

### 2.5 YY2 Inhibits EAC Proliferation by Promoting FOXRED1 Transcription

**Figure 5:**
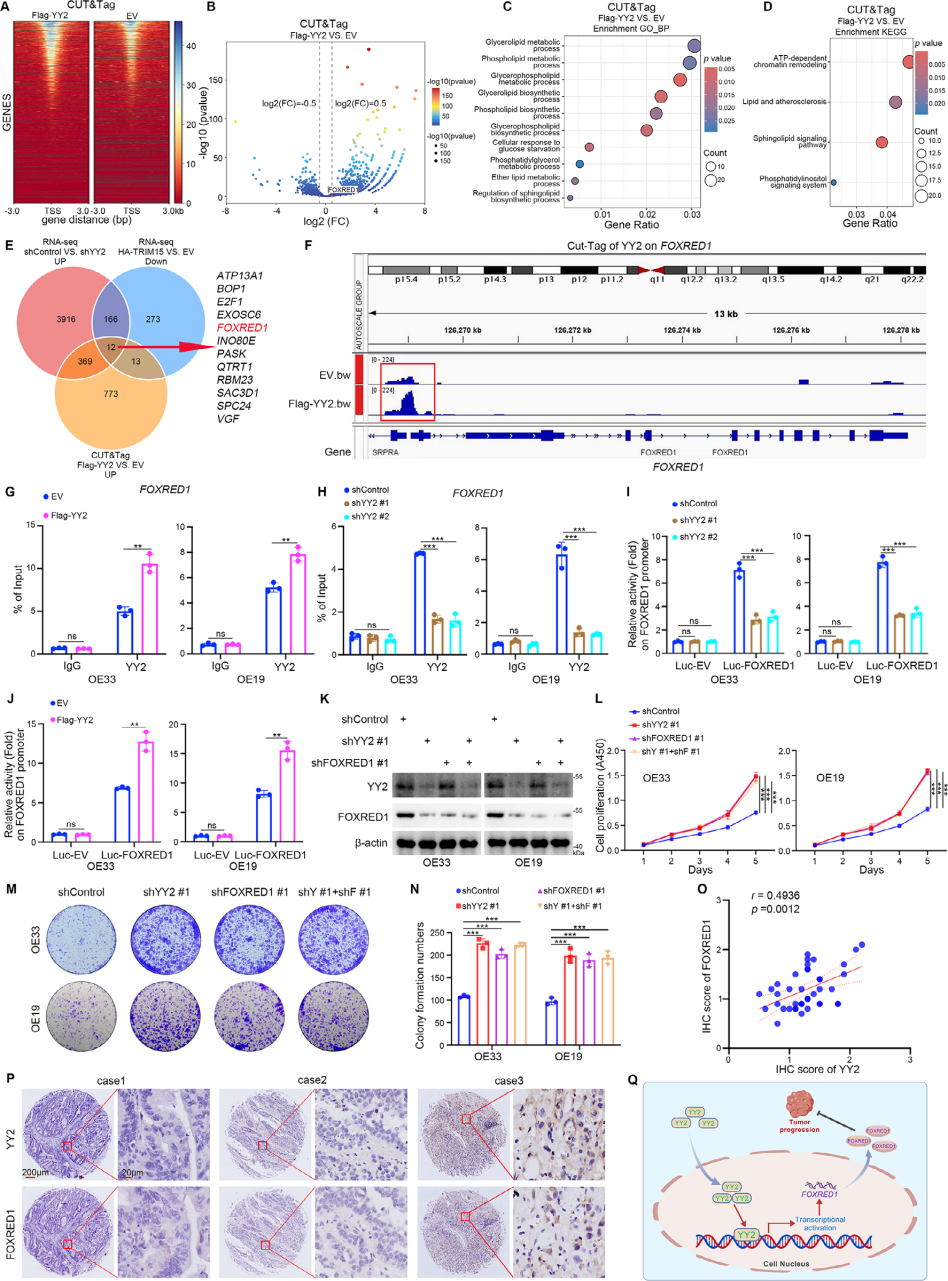
YY2 inhibits EAC proliferation by promoting *FOXRED1* transcription. A–D) The CUT&Tag sequencing analysis after transfection with EV or Flag‐YY2 for 48 h. Heat map (A), bubble map (B), GO‐BP (C), and KEGG (D) enrichment analyses of the CUT&Tag sequencing result. *p* values as indicated. E) Venn diagram of intersecting rows of the CUT&Tag sequencing result for YY2 and the RNA‐seq results for TRIM15 and YY2. F) The CUT&Tag of YY2 showed the binding peak of YY2 in the promoter region of *FOXRED1*. G and H) EAC cells were transfected with Flag‐YY2 or shYY2 plasmids for 48 or 72 h. Cells were harvested for ChIP‐qPCR analysis by using the IgG or YY2 antibody. Data presented as mean ± SEM with three replicates. ns, not significant; ^**^ *p* < 0.001; ^***^ *p* < 0.001. I and J) EAC cells were transfected with Flag‐YY2 or shYY2 plasmids for 48 or 72 h. Cells were harvested for a dual luciferase assay. Data presented as mean ± SEM with three replicates. ns, not significant; **, *p* < 0.001; ***, *p* < 0.001. K–N) EAC cells were transfected with shFOXRED1, shYY2 plasmids for 72 h. Cells were collected for Western blot analysis (K), CCK‐8 assay (L), and colony formation assay (M and N). Data presented as mean ± SEM with three replicates. ^**^ *p* < 0.001; ^***^ *p* < 0.001. O and P) The EAC tissue microarray was analyzed by IHC staining by using the anti‐YY2 or anti‐FOXRED1 antibody. *p* values as indicated. Q) The model diagram of YY2 inhibits EAC progression by promoting *FOXRED1* transcription.

Studies have demonstrated that YY2 is involved in the regulation of tumor progression as a transcription factor.^[^ ^26^ ^]^ To investigate the mechanisms by which YY2 regulates lipid metabolism and cell proliferation in EAC, we performed CUT&Tag analysis by overexpressing YY2 in OE33 cells. The results of CUT&Tag heatmaps (**Figure** **5**A), binding peaks (Figure S7A, Supporting Information), and bubble plots (Figure 5B) indicated that multiple genes were regulated by YY2 overexpression. The GO‐BP and KEGG enrichment analyses of CUT&Tag showed that YY2 overexpression in EAC cells regulated multiple lipid metabolism‐related pathways (Figure 5C and 5D). To further investigate the downstream genes co‐regulated by TRIM15 and YY2, we cross‐analyzed the CUT&Tag result of YY2 with the RNA‐seq results of TRIM15 and YY2, identifying twelve co‐regulated genes: *ATP13A1*, *BOP1*, *E2F1*, *EXOSC6*, *FOXRED1*, *INO80E*, *PASK*, *QTRT1*, *RBM23*, *SAC3D1*, *SPC24*, and *VGF* (Figure 5E). Mitochondria are the energy metabolism centers of the cell and are closely related to lipid and glucose metabolism.^[^ ^27^ ^]^ Combining GO‐BP and KEGG enrichment analyses from the RNA‐seq results of HFD transplantation tumors (Figure 1F,G), TRIM15 (Figure 2F,G), and YY2 (Figure 4B,C) in EAC cells, we found that lipid metabolism and energy metabolism are important processes in the progression of obesity‐associated EAC. Interestingly, previous studies have shown that FAD‐dependent oxidoreductase domain‐containing protein 1 (FOXRED1), a key factor regulating mitochondrial structure, is closely associated with energy conversion within mitochondria.^[^ ^28^ ^]^ Additionally, FOXRED1 is involved in regulating colorectal cancer progression as a tumor suppressor gene,^[^ ^29^ ^]^ but its biological function in EAC is unknown. Therefore, we focused on the *FOXRED1* gene.

The CUT&Tag data of YY2 demonstrated that YY2 protein has a binding site on the gene promoter of FOXRED1, with the binding peak higher in the YY2 overexpression group relative to the control group (Figure 5F). The results of ChIP‐qPCR and dual luciferase assays indicated that YY2 overexpression promoted the binding of YY2 to *FOXRED1* (Figure 5G–J). In addition, through the corresponding procedures on the JASPAR website (https://jaspar.elixir.no/), we predicted the binding motif of YY2 on the *FOXRED1* promoter and designed corresponding *FOXRED1* promoter wild‐type (*FOXRED1* ^WT^‐Luc) and mutant (*FOXRED1* ^Mut^‐Luc) plasmids of the *FOXRED1* promoter (Figure S7B, Supporting Information). Luciferase reporter analyses performed after transfection of the *FOXRED1* ^WT^‐Luc or *FOXRED1* ^Mut^‐Luc plasmids showed that altering the level of YY2 affected the luciferase activity of the *FOXRED1* ^WT^‐Luc promoter but not the *FOXRED1* ^Mut^‐Luc promoter (Figure S7C,D, Supporting Information). Further analysis showed that knockdown of YY2 in EAC cells decreased the protein and mRNA levels of FOXRED1 (Figure S8A,B, Supporting Information). In contrast, YY2 overexpression produced the opposite results (Figure S8C,D, Supporting Information). Furthermore, we found that knockdown of FOXRED1 in EAC cells significantly enhanced cell proliferation and colony formation (Figure S8E–H, Supporting Information). Conversely, FOXRED1 overexpression produced the opposite results (Figure S8I–L, Supporting Information). Additionally, we observed that simultaneous overexpression of YY2 and knockdown of FOXRED1 in EAC cells counteracted the inhibitory effect of YY2 overexpression alone on cell proliferation and colony formation (Figure 5K–N; SM–O, Supporting Information). Interestingly, by IHC analysis, we found that the expression of YY2 and FOXRED1 was lower in tumors of the HFD group compared to the ND group, whereas knockdown of TRIM15 elevated the expression of YY2 and FOXRED1 (Figure S9A–D, Supporting Information). IHC staining of tissue microarrays demonstrated a positive correlation between YY2 and FOXRED1 expression in EAC tissues (*r* = 0.4936, *p* = 0.0012) (Figure S5O,P, Supporting Information). These results suggest that YY2 inhibits EAC proliferation by activating *FOXRED1* transcription (Figure 5Q).

### 2.6 TRIM15 Dysregulates Glycerophospholipid Metabolism in EAC Cells by Inhibiting FOXRED1 Expression

**Figure 6:**
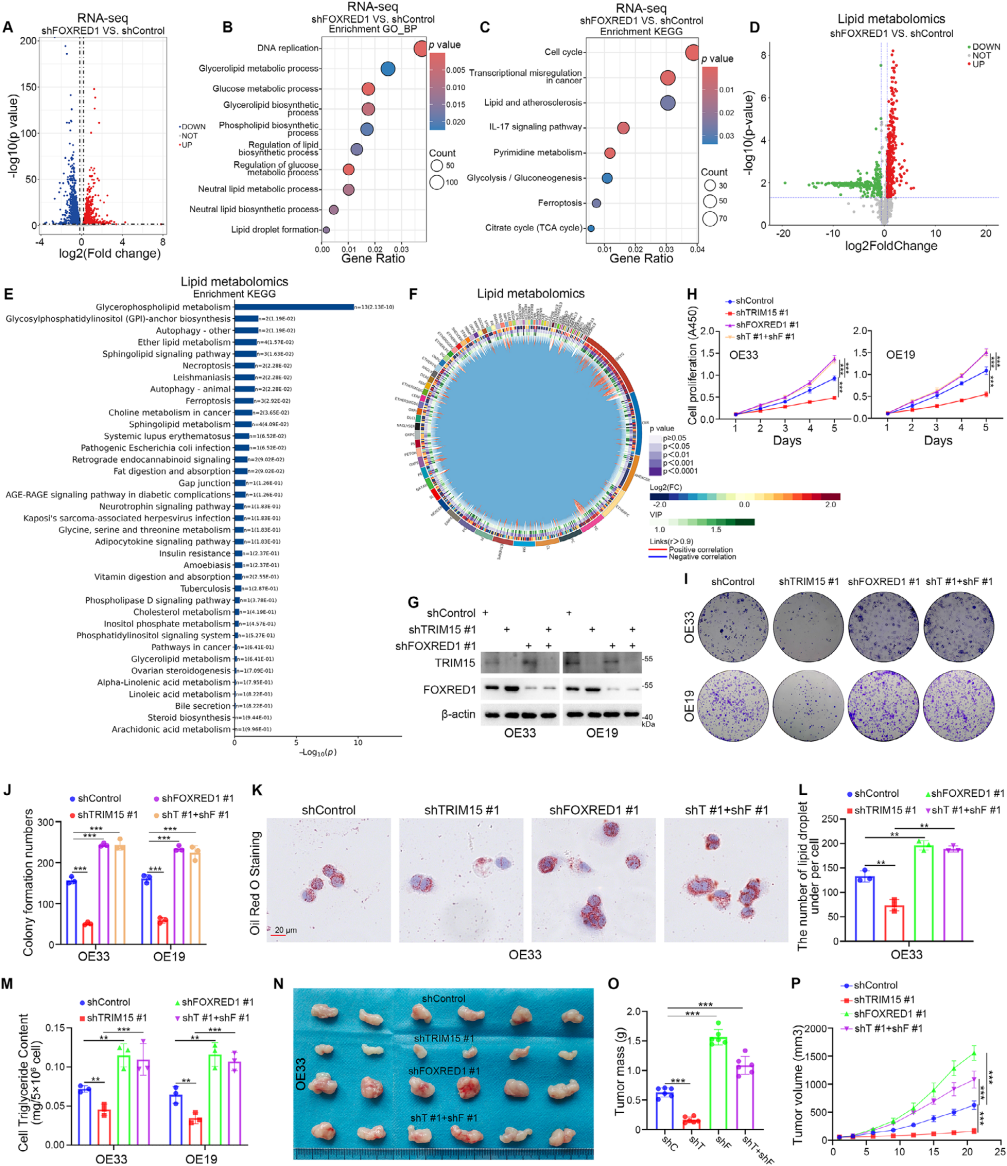
TRIM15 dysregulates lipid and energy metabolism by inhibiting FOXRED1. A–D) The transcriptome sequencing analysis of OE33 cells after transfection with shFOXRED1 or shControl plasmids for 72 h. Volcano map (A), GO‐BP (B), and KEGG (C) enrichment analysis of the RNA‐seq data after knockdown of FOXRED1 in OE33 cells. *p* values as indicated. D–F) The lipid metabolomics analysis of OE33 cells after transfection with shControl or shFOXRED1 plasmids for 48 h. Volcano map (D), KEGG enrichment analysis (E), Circos chord plot (From the outside to the inside are the names of the lipid molecules, classification, multiplicity of differences, *p*‐value, OPLS‐DA VIP, and correlation linkage (positive correlation in red, negative correlation in blue)) (F). *p* values as indicated. G–M) EAC cells were transfected with shTRIM15 or/and shFOXRED1 plasmids for 72 h. Cells were collected for Western blot analysis (G), CCK‐8 assay (H), colony formation assay (I and J), Oil Red O Staining (K and L), and cellular triglyceride assay (M). Data presented as mean ± SEM with three replicates. ^**^ *p* < 0.001; ^***^ *p* < 0.001. N–P) OE33 cells were transfected with shTRIM15 or/and shFOXRED1 plasmids for 72 h. After puromycin selection, these cells were subcutaneously injected into the nude mice. Tumor image (N), tumor mass (O) and tumor growth curve (P). Data presented as mean ± SEM with six replicates. ***, *p* < 0.001.

To explore whether FOXRED1 is involved in lipid metabolism in EAC cells, we knocked down FOXRED1 in OE33 cells and performed RNA‐seq analysis (**Figure** **6**A). The GO‐BP (Figure 6B) and KEGG (Figure 6C) enrichment analyses indicated that FOXRED1 knockdown significantly dysregulated lipid and glucose metabolism‐related pathways. Thus, we performed lipid metabolomics analysis by knocking down FOXRED1 in OE33 cells. The PCA and PLS‐DA analyses in lipid metabolomics for FOXRED1 showed stable and reliable results (Figure S10A–D, Supporting Information). The volcano plots (Figure 6D) and heatmap (Figure S10E, Supporting Information) indicate that multiple lipid abundances were affected. The Circos chord plot results of FOXRED1 lipid metabolomics show a strong association between the different lipids (Figure 6F). Knockdown of FOXRED1 increased the abundance of multiple lipids, including PA, PC, PG, and TG in EAC cells (Figure S10F–K, Supporting Information). Interestingly, we found that knockdown of FOXRED1 and overexpression of YY2 in EAC cells were both most significantly associated with Glycerophospholipid metabolism enrichment by KEGG enrichment analysis of the lipid metabolomics (Figures 4E and 6E). Meanwhile, the results of GO‐BP and KEGG enrichment analyses suggest that Glycerophospholipid metabolism is an important process for TRIM15, YY2, and FOXRED1 in EAC progression (Figures 1, 2, 4, 5, 6, and 6B,C). In addition, high levels of PCs, a major component of the phospholipid bilayer membrane, are required for tumor cell growth and proliferation.^[^ ^30^ ^]^ We then found that knockdown of TRIM15 in EAC cells reduced the abundance of PCs in the cells, whereas simultaneous knockdown of TRIM15 and YY2/FOXRED1 in the cells reversed the PCs‐reducing effect of knockdown of TRIM15 alone (Figure S11A–F, Supporting Information). Furthermore, addition of GPLs ((PCs (10 µg mL^−1^), PEs (10 µg mL^−1^), LysoPCs (10 µg mL^−1^), and LysoPAs (10 µg mL^−1^)) to EAC cells attenuated the inhibitory effect of knockdown of TRIM15 or overexpression of FOXRED1 on cell proliferation (Figure S11G–J, Supporting Information).

Glucose and lipid metabolism are critical for mitochondrial energy conversion.^[^ ^31^ ^]^ Lipid metabolism is closely linked to glucose metabolism, and increased glycolysis may redirect carbon flux toward increased lipogenesis.^[^ ^32^ ^]^ Dysregulation of glucose metabolism leads to an abnormal increase in lipid synthesis, further leading to dysregulation of lipid metabolism.^[^ ^33^ ^]^ Meanwhile, FOXRED1 plays an important role as a key factor involved in mitochondrial energy conversion.^[^ ^28^ ^]^ As expected, we observed that FOXRED1 knockdown resulted in increased glucose consumption rate, lactate production rate, and extracellular acidification rate (ECAR) but decreased the oxygen consumption rate (OCR) in EAC cells (Figure S12A–F, Supporting Information), while FOXRED1 overexpression yielded the opposite results (Figure S12G–L, Supporting Information). In contrast, TRIM15 knockdown inhibited the glycolytic process, decreased ECAR, but increased OCR in EAC cells (Figure S12M–R, Supporting Information), whereas TRIM15 overexpression increased the glycolytic process (Figure S12S–X, Supporting Information). In addition, we found that knockdown of TRIM15 in EAC cells decreased the cellular ATP levels, while simultaneous knockdown of FOXRED1 counteracted this effect (Figure S13A,B, Supporting Information). Complex I of the human mitochondrion is the most abundant enzyme in the respiratory chain, consisting mainly of a P module, a Q module, and an N module.^[^ ^34^ ^]^ Consistent with previous studies,^[^ ^28^ ^]^ knockdown of FOXRED1 in EAC cells resulted in a reduction of Complex I‐associated subunit levels (Figure S13C, Supporting Information). Concurrently, we found that knockdown of both TRIM15 and FOXRED1 (Figure S13D, Supporting Information) in EAC cells counteracted the inhibitory effect of TRIM15 knockdown alone on cell proliferation and colony formation (Figure 6G–J; Figure S13E–G, Supporting Information). Furthermore, we found that concomitant knockdown of TRIM15 and FOXRED1 counteracted the decreased effects of single TRIM15 knockdown on TG and lipid droplet levels (Figure 6K–M). Similar results were obtained in animal experiments (Figure 6N–P; Figure S13H–I, Supporting Information). These results indicate that TRIM15 dysregulates glycerophospholipid and energy metabolism by inhibiting FOXRED1 expression, thereby promoting EAC progression.

### 2.7 The TRIM15/YY2/FOXRED1 Axis Regulates the Sensitivity of EAC Cells to Ferroptosis

**Figure 7:**
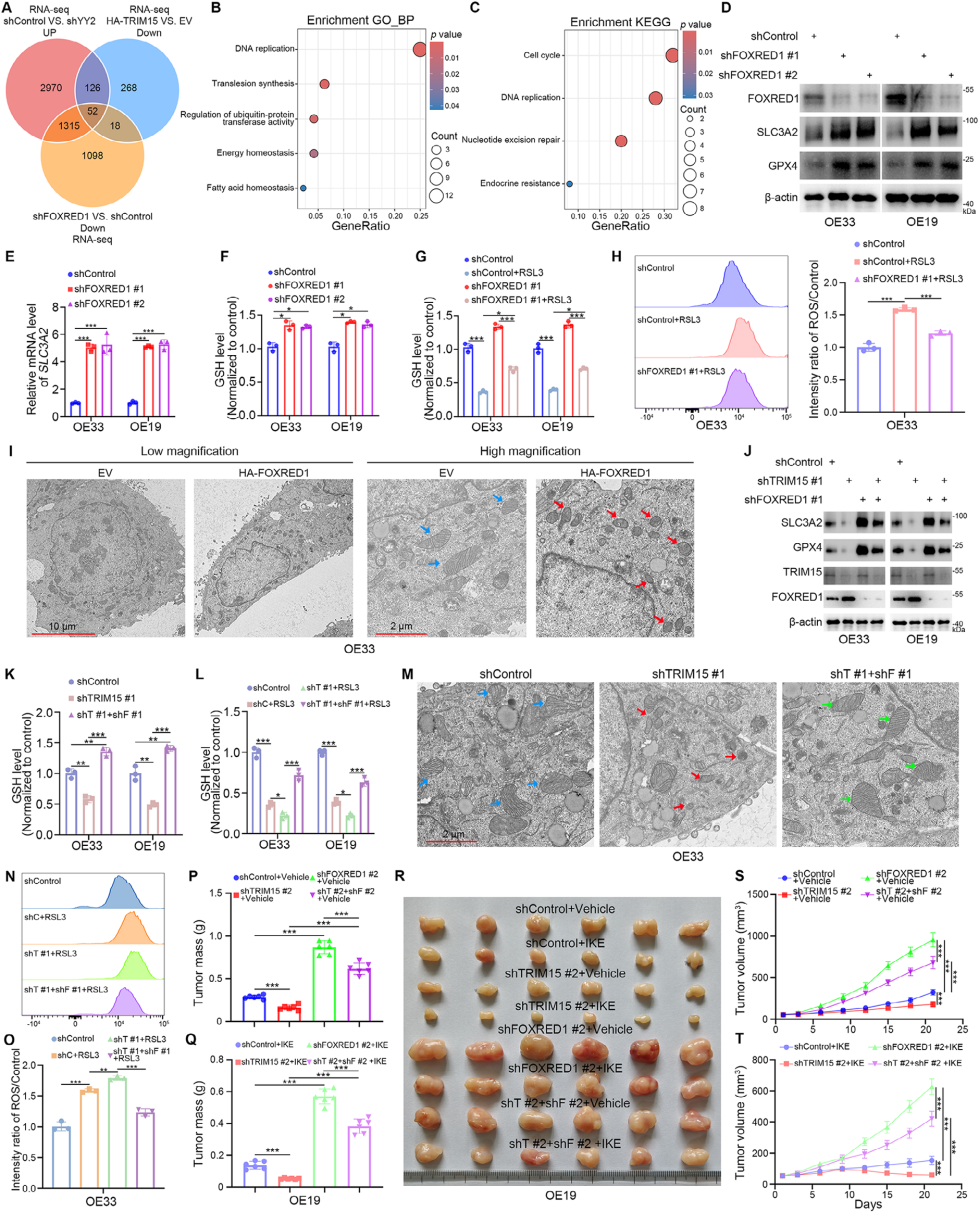
The sensitivity of EAC cells to ferroptosis is regulated by the TRIM15/YY2/FOXRED1 axis. A–C) Venn diagram (A), GO‐BP(B), and KEGG (C) enrichment analyses of intersecting rows of the RNA‐seq results for TRIM15, YY2, and FOXRED1. D and E, EAC cells were transfected with shFOXRED1 plasmids for 72 h. Cells were collected for Western blot analysis (D), RT‐qPCR analysis (E). Data presented as mean ± SEM with three replicates. ^***^ *p* < 0.001. F–H) EAC cells were transfected with shFOXRED1 plasmids for 48 h, and treated with RSL3 (1 µM) for another 24 h. Then, cells were collected for intracellular GSH levels assay (F and G), lipid ROS levels assay (H). Data presented as mean ± SEM with three replicates. ^*^ *p* < 0.05; ^***^ *p* < 0.001. I, Transmission electron microscopy images of mitochondria in OE33 cells overexpressing FOXRED1. Blue arrows, mitochondria with obvious cristae; red arrows, shrunken mitochondria. J‐O, EAC cells were transfected with shTRIM15 or/and shFOXRED1 plasmids for 48 h, and treated with RSL3 (1 µM) for another 24 h. Then, cells were collected for Western blot analysis (J), intracellular GSH levels assay (K and L), lipid ROS levels assay (N and O), and Transmission electron microscopy images (M) (Blue arrows, mitochondria with obvious cristae; red arrows, shrunken mitochondria; green arrows, mitochondria with obvious cristae). Data presented as mean ± SEM with three replicates. ^*^ *p* < 0.05; ^**^ *p* < 0.01; ^***^ *p* < 0.001. P–T) OE19 cells were transfected with shTRIM15 and shFOXRED1 plasmids for 72 h. After puromycin selection, these cells were subcutaneously injected into the nude mice, IKE (50 mg kg^−1^, once every other day) or vehicle was administered through intraperitoneal injection. Tumor mass (P and Q), tumor image (R), and tumor growth curve (S and T). Data presented as mean ± SEM with six replicates. ^***^ *p* < 0.001.

The above results indicated that the TRIM15/YY2/FOXRED1 axis contributed to EAC proliferation. Currently, no small molecule inhibitors targeting TRIM15 have been developed, limiting our findings’ clinical application. However, we discovered a significant correlation between the TRIM15/YY2/FOXRED1 axis and the Ferroptosis pathway by KEGG enrichment (Figures 1, 2, 4, and 6C,E). Furthermore, ferroptosis is driven by iron‐dependent phospholipid peroxidation.^[^ ^15^ ^]^ Biological lipid metabolism regulates ferroptosis by controlling phospholipid peroxidation and several other cellular processes relevant to phospholipid peroxidation.^[^ ^15^, ^16^ ^]^ Notably, lipidomic statistical analysis identified PCs and PEs as suppressors of cell ferroptosis.^[^ ^35^, ^36^ ^]^ Studies have shown that mitochondrial structural and functional abnormalities are closely associated with ferroptosis.^[^ ^14^ ^]^ FOXRED1 plays an important role in mitochondrial structure and function.^[^ ^28^, ^37^ ^]^ Then, we intersected the RNA‐seq results of *TRIM15*, *YY2*, and *FOXRED1*, and found that 52 genes were co‐regulated (**Figure** **7**A). The GO‐BP (Figure 7B) and KEGG (Figure 7C) enrichment analyses showed that the co‐regulated genes were significantly associated with the DNA replication, the energy homeostasis, the fatty acid homeostasis, the Cell cycle, and the Endocrine resistance pathways. Additionally, our analysis of FOXRED1 RNA‐seq data revealed that FOXRED1 knockdown in OE33 cells upregulated *SLC3A2* mRNA levels (Figure S14A, Supporting Information) and that KEGG enrichment analysis identified significant dysregulation of the mTOR signaling pathway (Figure S14B, Supporting Information). Previous studies have reported that c‐MYC, a key transcription factor in the mTOR pathway, is involved in the regulation of *SLC3A2*.^[^ ^38^, ^39^ ^]^ To investigate FOXRED1‐mediated regulation of the mTOR pathway, functional validation in EAC cells showed that FOXRED1 knockdown had no effect on total mTOR protein levels but significantly increased phosphorylated mTOR (p‐mTOR) and c‐MYC protein abundance (Figure S14C, Supporting Information). FOXRED1, as a key accessory factor of mitochondrial complex I, is crucial for maintaining mitochondrial functional homeostasis.^[^ ^28^ ^]^ Previous studies have established that metabolic disorders in mitochondrial Complex I play a critical role in promoting malignant tumor progression by activating the mTOR pathway.^[^ ^40^, ^41^ ^]^ Subsequently, we demonstrated that knocking down FOXRED1 promoted the activation of the mTOR/c‐MYC pathway by downregulating the expression of mitochondrial Complex I subunits (NDUFS2, NDUFB9) (Figure S14D,E, Supporting Information). We further found that knocking down FOXRED1 reduced the NAD^+^/NADH ratio (Figure S14F, Supporting Information) and increased glucose uptake (Figure S14H, Supporting Information). However, supplementation with the NAD^+^ precursor nicotinamide (2 mM) reversed the effects of FOXRED1 knockdown on the decrease in intracellular NAD^+^/NADH ratio (Figure S14G, Supporting Information), the increase in glucose uptake (Figure S14H, Supporting Information), and the activation of the mTOR/c‐MYC pathway (Figure S14I, Supporting Information). Additionally, analysis of public data from the NCBI SRA database (SRX11375739 and SRX095393) revealed a potential binding of c‐MYC to the *SLC3A2* promoter region (Figure S14J, Supporting Information). Further functional experiments demonstrated that c‐MYC knockdown in EAC cells reduced *SLC3A2* mRNA expression (Figure S14K, Supporting Information). Importantly, ChIP‐PCR assays confirmed that c‐MYC knockdown significantly attenuated its binding to the *SLC3A2* promoter, whereas FOXRED1 knockdown enhanced this binding interaction, an effect that was completely abrogated by the mTOR inhibitor Everolimus (Figure S14L, Supporting Information). Additionally, the FOXRED1 knockdown‐induced upregulation of SLC3A2 was reversed by concurrent c‐MYC knockdown or Everolimus treatment (Figure S14M,N, Supporting Information). Collectively, these results indicate that FOXRED1 modulates the mTOR/c‐MYC signaling axis by influencing mitochondrial homeostasis, thereby regulating SLC3A2 expression.

SLC3A2, a key member of system XC^−^, inhibits ferroptosis under oxidative stress conditions by transporting cystine into cells for the synthesis of the major antioxidant glutathione (GSH), which further affects GPX4 activity.^[^ ^42^ ^]^ Subsequently, we verified that in EAC cells, FOXRED1 was negatively correlated with the levels of SLC3A2 and GPX4 (Figure 7D–E). Concomitantly, FOXRED1 knockdown in EAC cells resulted in increased GSH levels (Figure 7F). We found that FOXRED1 knockdown attenuated the inhibitory effect of the ferroptosis‐inducing compounds (RSL3 and IKE) on GSH levels (Figure 7G; Figure S16A, Supporting Information), as well as Reactive Oxygen Species (ROS) production (Figure 7H) in EAC cells. Similarly, transmission electron microscopy results demonstrated that overexpression of FOXRED1 induced profound changes in OE33 cells, specifically a reduction in mitochondrial volume, heightened density of bilayer membranes, and disappearance of mitochondrial ridges (Figure 7I). Additionally, we found that YY2 knockdown in EAC cells increased GSH levels while attenuating the inhibitory effect of RSL3 on GSH (Figure S15A, Supporting Information). Nevertheless, TRIM15 knockdown in EAC cells decreased the levels of SLC3A2 and GPX4 (Figure S15B, Supporting Information). Transmission electron microscopy results demonstrated that TRIM15 knockdown in OE33 cells exhibited the same features as FOXRED1 overexpression, including a reduction in mitochondrial volume and disappearance of mitochondrial ridges (Figure S15C, Supporting Information). TRIM15 knockdown exacerbated the inhibition of GSH levels by RSL3 and IKE (Figures S15D and S16B, Supporting Information), as well as exacerbated RSL3‐induced ROS production (Figures S15E and S14F, Supporting Information) in EAC cells. Furthermore, we sought to investigate the regulatory relationship among TRIM15, FOXRED1, and ferroptosis in EAC cells. Our findings indicate that simultaneous knockdown of TRIM15 and FOXRED1 attenuated the potentiating effect of single TRIM15 knockdown on EAC cell sensitivity to RSL3 and IKE (Figure 7K–O; Figure S16C, Supporting Information). Meanwhile, we found that overexpression of SLC3A2 in EAC cells counteracted the inhibitory effect of knockdown of TRIM15 on GSH levels (Figure S15G,H, Supporting Information). Furthermore, to elucidate the regulatory effect of TRIM15 on GPX4, we demonstrated that GPX4 overexpression in EAC cells effectively rescued the enhanced inhibitory effect of RSL3 on cellular GSH levels caused by TRIM15 knockdown (Figure S15I,J, Supporting Information), while simultaneously attenuating intracellular ROS accumulation (Figure S15K,L, Supporting Information). Interestingly, we found that the addition of GPLs intervention in EAC cells attenuated the inhibitory effect of overexpression of YY2/FOXRED1 or knockdown of TRIM15 in combination with RSL3 on cell viability (Figure S16D–F, Supporting Information), and also attenuated the effect of knockdown of TRIM15 in combination with RSL3 in accelerating the generation of ROS in the cells (Figure S16G,H, Supporting Information). In vivo experiments showed that IKE treatment exacerbated the inhibition of EAC cell proliferation caused by TRIM15 knockdown, and proved that concomitant TRIM15 and FOXRED1 knockdown in EAC cells attenuated the enhancing effect of TRIM15 knockdown alone on IKE sensitivity (Figure 7P–T). Collectively, our results suggest that the TRIM15/YY2/FOXRED1 axis modulates the sensitivity to ferroptosis‐inducing compounds.

## 3 Discussion

Obesity and metabolic syndrome are major causes of cancer development.^[^ ^43^ ^]^ Among these obesity‐induced cancers, the incidence of EAC is significantly and positively correlated with obesity.^[^ ^1^ ^]^ Targeted lipid metabolism therapy is effective in reducing the risk of many cancers, including EAC.^[^ ^6^, ^44^ ^]^ Visceral fat in obese people releases large amounts of pro‐inflammatory cytokines (TNF‐α, IL‐6, and adipokines, etc.), which further induce tumorigenesis.^[^ ^45^, ^46^, ^47^ ^]^ Notably, studies have demonstrated that the inflammatory factors TNF‐α and IL‐22 induce elevated TRIM15 expression in cells.^[^ ^12^, ^48^ ^]^ Few studies have addressed obesity‐associated lipid metabolic disorders in EAC. We therefore performed RNA‐seq on subcutaneous EAC tumors from nude mice fed a HFD or ND. Among the top five significantly differentially expressed genes (DEGs), TRIM15 ranked first. Previous studies have clearly demonstrated that inflammatory factors promote the transcriptional upregulation of TRIM15 via the NF‐κB signaling pathway.^[^ ^12^ ^]^ As members of the serine protease inhibitor family, SERPINB4^[^ ^49^ ^]^ and SERPINB3^[^ ^50^ ^]^ primarily function as proto‐oncogenes to drive tumor cell proliferation; however, their association with inflammatory factors remains unclear. SAMSN1 is primarily associated with the induced differentiation of macrophages.^[^ ^51^ ^]^ Although MEFV is mainly involved in inflammatory regulation,^[^ ^52^ ^]^ its role in tumors has been poorly studied and remains ambiguous. Therefore, we focused on TRIM15 for subsequent investigations. Consistently, HFD mice had elevated plasma lipids and inflammatory factors. These inflammatory factors promoted *TRIM15* transcription via NF‐κB pathway activation, ultimately driving *TRIM15* upregulation in EAC cells.

TRIM is a family of proteins belonging to the E3 ubiquitin ligases that play a pivotal role in lipid metabolism, associated with a multitude of diseases, including cancer.^[^ ^53^ ^]^ TRIM8 exacerbates hepatic metabolic disorders by targeting TAK1 and activating the JNK/p38 and NF‐κB signaling pathways, leading to the development of HFD‐associated nonalcoholic fatty liver disease (NAFLD).^[^ ^54^ ^]^ Similarly, enhanced TRIM67 expression may contribute to the progression of obesity‐induced NAFLD by activating hepatic inflammation to disrupt lipid metabolic homeostasis.^[^ ^17^ ^]^ Furthermore, TRIM67 plays a role in the hypothalamus function in regulating energy homeostasis through the NF‐κB pathway.^[^ ^55^ ^]^ Meanwhile, TRIM16,^[^ ^56^ ^]^ TRIM31,^[^ ^18^, ^57^ ^]^ TRIM59,^[^ ^58^ ^]^ TRIM38,^[^ ^59^ ^]^ and TRIM56^[^ ^60^ ^]^ play key roles in the development of HFD‐induced NAFLD/NASH. Interestingly, specific knockdown of TRIM28 in adipocytes leads to the development of obesity, a process involving alterations in lipolysis and TG metabolism.^[^ ^19^ ^]^ It is clear that the TRIM family is involved in the progression of many obesity‐associated diseases by dysregulating lipid metabolism, but the biological function of the TRIM family in obesity‐induced EAC is unknown. Combining the previous studies and the RNA‐seq results in our HFD mouse model, we focused on *TRIM15*.

It is well established that cancer cells exhibit altered cellular metabolism and energy production.^[^ ^61^ ^]^ Multiple pathways and processes alter cellular utilization of metabolites and chemicals, promoting abnormal cell replication, tumor formation, and immune escape.^[^ ^62^ ^]^ Concurrently, obesity can induce subtle alterations in the microenvironment, leading to pronounced cell signaling involving lipid compounds, ultimately facilitating malignant tumor progression.^[^ ^63^ ^]^ Dysfunction of TRIM15 protein has been associated with the progression of various tumors, including esophageal squamous carcinoma,^[^ ^64^ ^]^ non‐small cell lung cancer,^[^ ^65^ ^]^ gastric adenocarcinoma^[^ ^66^ ^]^ and pancreatic cancer.^[^ ^13^ ^]^ In previous studies, we demonstrated that TRIM15 dysregulates lipid metabolism to promote pancreatic cancer invasion and metastasis.^[^ ^10^ ^]^ Furthermore, we found that TRIM15 could regulate the sensitivity of Hepatocellular carcinoma cells to TKIs by forming a regulatory loop with the AKT/FOXO1 axis and LASP1.^[^ ^11^ ^]^ Differently, in our present study, we identified TRIM15 as a key molecule in obesity‐associated EAC. Further studies revealed that TRIM15 promotes tumor progression by mediating the ubiquitination and subsequent degradation of YY2, disrupting lipid and energy metabolism regulation in EAC cells by inhibiting *FOXRED1* transcription. Notably, our lipid metabolomics results showed that alterations in YY2 and FOXRED1 significantly affect the abundance of various lipid metabolites in EAC cells, regulating tumor progression mainly through the glycerophospholipid metabolic pathway.

More important, lipid metabolism controls ferroptosis by regulating phospholipid peroxidation and several other cellular processes involved in phospholipid peroxidation.^[^ ^15^, ^16^ ^]^ Interestingly, we found that TRIM15 is involved in regulating the ferroptosis process in EAC cells. Ferroptosis is an iron‐dependent regulatory cell death, and ferroptosis induction therapy has emerged as a pivotal strategy in oncology.^[^ ^14^ ^]^ Developing small‐molecule drugs targeting the ferroptosis‐regulated pathway is of particular significance.^[^ ^14^, ^67^ ^]^ Increasing experimental evidence suggests that many metabolic pathways, including cellular respiration, lipid metabolism, and amino acid metabolism, contribute to ferroptosis through Lipid Reactive Oxygen Species (L‐ROS) production.^[^ ^14^, ^68^ ^]^ Meanwhile, Li et al. have shown that YY2 regulates ferroptosis in tumor cells through the SLC7A11/GSH synthesis axis.^[^ ^24^ ^]^ The system XC^−^ is a heterodimeric complex consisting of two integral membrane proteins, SLC3A2 and SLC7A11.^[^ ^69^ ^]^ SLC3A2 functions as a chaperone for SLC7A11, playing a pivotal role in maintaining the structural integrity of SLC7A11 and regulating its trafficking to the plasma membrane.^[^ ^69^, ^70^ ^]^ Specifically, system XC^−^ mediates the extracellular uptake of cystine, a rate‐limiting precursor for GSH synthesis, while concomitantly releasing glutamic acid into the extracellular milieu.^[^ ^71^ ^]^ GSH, a tripeptide antioxidant, is indispensable for protecting cells from oxidative stress and maintaining the delicate equilibrium of the cellular redox state.^[^ ^72^ ^]^ At the same time, as a GSH‐dependent peroxidase, the main function of GPX4 is to catalyze the reaction between GSH and lipid peroxides, reducing the toxic lipid peroxides to their corresponding alcohols while GSH is oxidized to GSSG.^[^ ^72^, ^73^, ^74^ ^]^ In our study, we discovered that the TRIM15/YY2/FOXRED1 axis regulates the expression of SLC3A2 through the mTOR/c‐MYC pathway, which in turn affects the expression of GPX4, and ultimately modulates the sensitivity of EAC cells to ferroptosis.

In summary, our study innovatively found that TRIM15 expression is upregulated in obesity‐associated EAC tumor tissues, emerging as a key molecule driving the proliferation stemming from obesity. We found that TRIM15 degraded YY2 protein, inhibiting FOXRED1 expression to promote EAC proliferation. Additionally, we elucidated the critical role of the TRIM15/YY2/FOXRED1 axis in dysregulating lipid metabolism, energy metabolism, and ferroptosis in EAC. In conclusion, our study provides new insights into the pathogenesis of obesity‐associated EAC and may provide ideal candidates for EAC drug development.

## 4 Experimental Section

### Cell Lines and Cultures

Esophageal adenocarcinoma (EAC) cell lines OE33 (Cat No. BH‐C232, Bohui Biological Technology), OE19 (Cat No. BH‐C247, Bohui Biological Technology), and 293T (Cat No. SNL‐015, Sunncell) cells were identified by STR. Cells were cultured in RPMI‐1640 (Gibco, USA) or DMEM (Gibco, USA) containing 10% fetal bovine serum (FBS) (Cat No. AC03L055, Shanghai Life‐iLab Biotech, China) and 1% penicillin‐streptomycin. Cultures were incubated at a constant temperature of 37 °C with 5% CO_2_ and tested periodically for mycoplasma‐free growth. EAC cells were treated with IL‐6 (50 ng mL^−1^, 48 h; Cat No. HY‑P7044, Med Chem Express), NF‐κB inhibitor (10 µM, 48 h; BAY‐11‐7082, Cat No. S1523, Beyotime), and Everolimus (1 µM, 24 h, Cat No. HY‐10218, Med Chem Express) for further studies. EAC cells were treated with glycerophospholipids (GPLs) (PC (10  µg mL^−1^, 48 h; Cat No. HY‐B2235, Med Chem Express), PE (10  µg mL^−1^, 48 h; Cat No. HY‐W250118, Med Chem Express), LysoPC (10  µg mL^−1^, 48 h; Cat No. HY‐139414, Med Chem Express), LysoPA (10  µg mL^−1^, 48 h; Cat No. HY‐157639A, Med Chem Express)) for further studies. EAC cells were treated with ferroptosis‐inducing compounds RSL3 (1 µM, 24 h; Cat No. HY‐100218A, Med Chem Express) and IKE (10 µM; Cat No. HY‐114481, Med Chem Express) for further studies.

### Transfection Plasmids

Plasmid cell transfection was performed by using Lipofectamine acetamide 2000 (Cat No. 11 668 019, Thermo Fisher Scientific, USA) according to the manufacturer's instructions when the cells were grown to the appropriate density in a culture plate or dish. In addition, stably transfected cells were obtained through ≈3 weeks of puromycin screening. The sequences of all short hairpin RNA (shRNAs) are listed in Table S1 (Supporting Information).

### Immunohistochemistry (IHC)

EAC tumor tissues were collected from the Department of Thoracic Surgery, The Second Xiangya Hospital of Central South University, and informed consent was signed by the patients. The study was approved by the Clinical Research Ethics Committee of The Second Xiangya Hospital of Central South University (NO. LYEC2024‐K0220). Esophageal and gastric cardia adenocarcinoma tissue microarrays (Cat No. D046Ca01, China) were purchased from Bioaitech. Immunohistochemical analysis was performed using specific antibodies. IHC scores were determined by two independent pathologists who were unaware of the patient's data. The IHC score was calculated based on the intensity of cell staining (0/1/2/3) and the positivity rate (0‐100% positive cells). The IHC score was equal to the product of the intensity of staining and the positivity rate. Antibodies used for IHC are shown in Table S2 (Supporting Information).

### CCK‐8 Assay

EAC cells with different treatments were seeded into 96‐well plates (3000 cells per well). The next day, after washing once with PBS, CCK‐8 reagent (Cat No. AC11L054, Shanghai Life‐iLab Biotech, China, 20 µL) was added to each well, and the optical absorbance at 450 nm was measured by using a microplate reader for 5 consecutive days.

### Colony Formation Assay

EAC cells treated with different treatments were inoculated into 6‐well plates (1000 cells per well) by cell counting. After 2 weeks, the cells were washed twice with PBS, fixed with 4% paraformaldehyde for 20 min, then stained with 1% crystal violet staining solution (Cat No. C0121, Beyotime) for 30 min, and finally washed three times with PBS. After drying, the cells were photographed and counted.

### Oil Red O Staining

Cells or frozen sections of tissues were fixed in 4% paraformaldehyde at room temperature for 20 min. A 70% ethanol solution was used to rinse once, and then oil red staining solution was added for 8 min. 70% ethanol solution was used to wash again and rinsed under running water for 30 s. Hematoxylin staining solution was used for 2 min for glycerol gelatin sealing. Finally, the samples were analyzed by microscopic observation.

### Quantitative real‐time PCR (RT‐qPCR)

Total RNA was extracted with TRIzol reagent (Cat No. AG21102, Accurate Biotechnology, Hunan, China) and reverse transcribed into complementary DNA using PrimeScript RT reagent kit (Cat No. AG11728, Accurate Biotechnology, Hunan, China). RT‐qPCR was performed using the TBGreen Fast qPCR Mix kit (Cat No. AG11701, Accurate Biotechnology, Hunan, China). β‐actin was used as an internal reference gene, and the 2^−ΔΔCt^ method was used to quantify the fold change. The primer sequences used for RT‐qPCR are shown in Table S3.

### Western Blotting

EAC cells after different treatments were collected, cells were washed twice with pre‐cooled PBS, and RIPA lysate (Cat No. P0013C, Beyotime) containing phosphatase inhibitor and protease inhibitor was added to the cells and lysed on ice for 30 min. Subsequently, the cell lysate was centrifuged at 12000 rpm for 15 min at 4 °C, and the supernatant was collected. The protein concentration of each group of cells was determined using a protein quantification kit (Cat No. P0012S, Beyotime). Protein lysates from each group of cells were separated by SDS‐PAGE, and the proteins were transferred to a PVDF membrane. Next, the protein‐laden PVDF membrane was incubated with a specific antibody (primary antibody) overnight. The next day, the PVDF membrane was washed three times with 1×TBST and then incubated with the secondary antibody for 2 h. The membrane was then washed three times with 1×TBST and then detected on the machine by enhanced chemiluminescence. Antibodies used for protein blotting are shown in Table S2 (Supporting Information).

### Co‐immunoprecipitation (Co‐IP)

Western/IP lysis buffer (Cat No. P0013, Beyotime) was added to the collected EAC cells and placed on ice for 30 min to fully lyse. Subsequently, the supernatant was collected by centrifugation at 12 000 rpm and 4 °C for 15 min. 80 µL of the supernatant was taken as INPUT, and the remaining supernatant was added with an appropriate amount of primary antibody and protein A/G agarose beads (Cat No. P2029, Beyotime) and rotated and incubated overnight at 4 °C. The next day, the beads were collected by centrifugation at 3000 rpm and washed six times with Western/IP lysis buffer. The proteins were separated by SDS‐PAGE gel electrophoresis after resuspension in 1×Sampling Buffer and boiling for 10 min, and transferred to PVDF membranes. The subsequent steps were consistent with Western blotting. Specific antibodies used for Co‐IP are listed in Table S2 (Supporting Information).

### Chromatin Immunoprecipitation (ChIP) Assay

Chromatin immunoprecipitation was performed using the ChIP Assay Kit (Cat No. P2078, Beyotime) according to the manufacturer's instructions. Briefly, cells were lysed, and chromatin was immunoprecipitated using protein A+G agarose/salmon sperm DNA and anti‐YY2 antibody or normal rabbit IgG, uncrosslinked for 4 h at 65 °C, and treated with 0.5 M EDTA, 1 M Tris (pH 6.5), and 20 mg mL^−1^ proteinase K. The immunoprecipitated chromatin was subjected to PCR using PrimeSTAR Max.

### Dual‐luciferase Reporter Assay

EAC cells were seeded into 6‐well plates (≈8 × 10^5^ cells per well). 24 h later, cells were cotransfected with the indicated shRNAs or overexpression vectors and Renilla luciferase expression vector (internal control) (pRL‐SV40, Promega). After 48 h, luciferase activity was measured using a dual‐luciferase assay system (Promega). The relative light units of luciferase were normalized to the corresponding Renilla luciferase activity.

### High‐Fat Diet (HFD) and Xenograft Nude Mouse Models

All animal experimental procedures were approved by the Ethics Committee of The Second Xiangya Hospital of Central South University (NO. 20 240 256). BALB/c‐nu nude mice (4‐week‐old, male) were purchased from SJA Laboratory Animal Company (Hunan, China) and kept under pathogen‐free conditions for one week before the experiment. The mice were divided into two groups: high‐fat diet (35.0% fat, 26.0% carbohydrate, and 26.0% protein) and normal diet (4.3% fat, 67.3% carbohydrate, and 19.2% protein).^[^ ^75^ ^]^ Mice fed the HFD and with body weights exceeding the mean + 2× SD of the ND group. After 8 weeks of feeding, EAC cells (5 × 10^6^) were dispersed in 100 µL of PBS and inoculated subcutaneously on the left dorsal side of nude mice, which were continued to be fed with the corresponding diets. 21 days later, all mice were executed after anesthesia (40 mg kg^−1^ pentobarbital sodium), and blood was collected from the retro‐orbital venous plexus and retained for assessment of metabolites or adipokines, and the tumor tissues were removed subcutaneously for subsequent experiments.

EAC cells in PBS (5 × 10⁷ cells/mL) and subcutaneously injected 100 µL of the suspension into the dorsum of nude mice. The subcutaneous tumor volume was measured every 3 days with calipers and estimated as follows: tumor volume (mm^3^) = (width)^2^ × length × 1/2. To evaluate the therapeutic efficacy of IKE in a subcutaneous tumor‐forming model in nude mice, mice were treated with IKE (dissolved in vehicle (10% DMSO, 40% PEG300, 5% Tween 80, and 45% saline) and intraperitoneal injection, 50 mg kg^−1^, once every other day) or vehicle starting on day 9 after inoculation with different groups of EAC cells. After 21 days, the nude mice were euthanized, and the tumor tissues were taken subcutaneously and weighed, followed by immunophenotyping by IHC. The corresponding antibodies are listed in Table S2 (Supporting Information).

### Lipid Metabolomics Analysis

Lipid metabolomics analysis was carried out by Shanghai Bioprofile Biotechnology (China). Briefly, 200 µL of pre‐cooled 75% methanol was added to EAC cells (1 × 10^7^ cells), vortexed evenly, and sonicated on ice for 15 min. Add 1 mL of pre‐cooled MTBE and vortex for 1 h in a refrigerator at 4 °C, continue sonicating on ice for 30 min. Add 100 µL of water, vortex for 1 min, and then place for 10 min. Centrifuge the cells at 14 000 g for 15 min at 4 °C, remove the supernatant, and air dry the precipitate. 200 µL SDT was added to the precipitate, and the protein concentration was quantified by the BCA method. The supernatant was taken, and the precipitate was air‐dried. Based on the amount of protein in the sample, the sample was reconstituted with 55 µL isopropanol/methanol (1/1, v/v), transferred to the injection bottle, centrifuged at 20 000 g for 20 min at 4 °C, and the supernatant was taken into the sample for analysis. Preparation of quality control (QC) samples: Aliquots of each group of samples were mixed as QC.

Chromatography‐Mass Spectrometry: The samples were placed in an autosampler at 4 °C throughout the analysis, and the samples were analyzed on a SHIMADZU‐LC30 Ultra‐High Performance Liquid Chromatography (UHPLC) system using an ACQUITY UPLC HSS C18 (2.1 × 100 mm, 1.9 µm) (Waters, Milford, MA, USA) column. Subsequently, detection was carried out in electrospray ionization (ESI) positive and negative ion modes, respectively. The samples were separated by UHPLC and analyzed by mass spectrometry using a Q Exactive Plus mass spectrometer (Thermo Scientific). Finally, the software MSDAIL (Version 4.0.9) was used for lipid identification and quantification, among other processes.

### CUT&Tag Analysis

CUT&Tag analysis was performed at Jiayin Biotechnology Ltd (Shanghai, China). Shortly, cell nuclei were isolated from the OE33 cells with overexpressing Flag‐YY2 or EV. The purified nuclei (≈5 × 10^5^) were washed and incubated with Concanavalin A‐coated magnetic beads. The purified nuclei were then resuspended in dig‐wash buffer (with a 1:50 dilution of YY2 antibody or IgG as a control) and incubated overnight at 4 °C on a rotary platform. The beads were then washed in dig‐wash buffer and incubated with a compatible secondary antibody. Protein A‐Tn5 transposome was added to the beads for further incubation after secondary antibody incubation. Tn5 cuts the DNA fragments around the nucleosomes. Adapter sequences were added. The cells were then resuspended in tagmentation buffer and incubated at 37 °C for 1 h. DNA was purified via phenol‐chloroform‐isoamyl alcohol extraction and ethanol precipitation. Sequencing was performed on the Illumina Novaseq 6000 (manufacturer's information) using the pe150 sequencing protocol.

### Statistical Analysis

All data were statistically analyzed using GraphPad Prism software (version 8.0). Statistical significance was determined using an unpaired Student's t‐test (for two groups) or one‐way/two‐way ANOVA with post‐hoc tests (for >2 groups). Data are presented as mean ± standard deviation (SD). All statistical details of the experiments are mentioned in the legend, and all tests were two‐sided; a *p*‐value<0.05 was considered statistically significant.

### Ethics Approval and Consent to Participate

The study was conducted in accordance with the principles of the Declaration of Helsinki. It was approved by the Clinical Research Ethics Committee of the Second Xiangya Hospital of Central South University (No. LYEC2024‐K0220).

## Conflict of Interest

The authors declare no conflict of interest.

## Author Contributions

H.W., C.Y., D.L., Pi.L., Z.Z., W.P., D.P., and H.S. performed methodology. H.W., C.Y., D.L., and P.L. performed formal analysis. H.W. wrote the original draft. H.W. and X.J. performed writing, reviewing, and editing. C.Y., X.X., and H.W. performed funding acquisition. X.X., H.W., and X.J. performed project administration and investigation.

## Supporting information



Supporting Information

Supporting Information

Supporting Information

Supporting Information

## Data Availability

The datasets used and/or analyzed during the current study are available from the corresponding author (**Xin Jin**, **E‐mail**: jinxinxy2@csu.edu.cn) on reasonable request. The sequencing data were deposited in the GEO public dataset (**GSE293844**).
