# Supplementary material for: Obesity‐Associated TRIM15 Promotes the Proliferation of Esophageal Adenocarcinoma Through the YY2/FOXRED1 Axis
Source: Adv Sci (Weinh). 2025 Nov 14;13(4):e17330. doi: 10.1002/advs.202417330 (PMC12822407; doi:10.1002/advs.202417330)
Supplement: Supplementary file 1 — Supporting Information [file ADVS-13-e17330-s001.docx]

**Supplementary Methods**

**Meta-analysis**

Following the guidelines outlined by the Preferred Reporting Items for Systematic Reviews and Meta-Analyses (PRISMA)[1], we conducted a comprehensive selection criteria and statistical analysis of the literature. We utilized the following search terms: “abdominal obesity (AO)” or “central adiposity” or “visceral obesity”, “esophageal adenocarcinoma (EAC)” or “oesophageal adenocarcinoma (OAC)” or “adenocarcinoma of the esophagus”, and “Barrett esophagus (BE)” or “Barrett's oesophagus (BO)” to search for relevant studies in Pubmed, Embase, and Cochrane Library databases up to November 2024.

Studies were included when the following criteria were fulfilled: (1) Case-control and cohort studies were chosen to assess the association between EAC/BE and obesity; (2) Accurate definition of EAC or BE with histological evidence; (3) Well-defined obesity estimated by (BMI, kg/m^2^); (4) Studies reported OR and weighted mean difference (WMD) with 95% CI; (5) Studies (not case reports or meta-analyses) including estimation risk and initial data.

**Plasmid Construction**

The shRNA and overexpression plasmids were constructed through a sequential workflow. First, 19-21 nt target-specific sequences were selected from the coding region of the gene of interest, followed by homology screening via NCBI BLAST to eliminate sequences with high similarity to non-target genes. Subsequently, complementary strands and a 4-10 nt loop sequence were designed to form a hairpin structure, with restriction enzyme sites incorporated at both ends of the shRNA sequence. Next, synthesized complementary oligonucleotides (Tsingke Biotechnology, China) were dissolved in 50 μL sterile ddH₂O, mixed in equal volumes, and annealed in a thermal cycler (95°C for 5 min, then cooled to 25°C at 0.1°C/s). For plasmid assembly, the shRNA vector (pLV3-U6-MCS-shRNA-CopGFP-Puro) or overexpression vector (PCDH-3×HA/Flag/His-puro-GFP) was digested with appropriate restriction enzymes (37°C, 3 h), purified via agarose gel electrophoresis, and ligated to annealed oligonucleotides at a 5:1 molar ratio using T4 DNA ligase (25°C, 3 h). Following ligation, the recombinant plasmids were transformed into CaCl₂-treated E. coli competent cells via heat shock (42°C, 90 s), recovered in antibiotic-free LB medium (37°C, 1 h), and plated on ampicillin-containing LB agar for overnight incubation (37°C). To screen positive clones, single colonies were cultured in ampicillin-LB broth (37°C, overnight), and plasmid DNA was extracted, restriction-digested, and verified by PCR. Confirmed clones were sequenced (Tsingke Biotechnology). Finally, validated clones were amplified in large-scale LB cultures (37°C, 12-16 h), and plasmids were purified using a maxi-prep kit, quantified spectrophotometrically, and stored at -20°C.

**Measurement of cell triglyceride (TG), phosphatidylcholine (PC) content, NAD^+^/NADH analysis and Glucose uptake assay**

Triglyceride Fluorometric Assay Kit (E-BC-F033, Elabscience Biotechnology Co.,Ltd. China) was used to measure the TG content in EAC cells. EAC cells (5×10^6^) were homogenised with 1 mL of Extraction Solution, centrifuged at 10,000 × g for 10 min, and the supernatant was taken for measurement. Analysis was conducted according to the instructions of the manufacturer.

Cellular phosphatidylcholine (PC) levels were measured by using a [Phosphatidylcholine Colorimetric Assay Kit](https://www.elabscience.cn/p-phosphatidylcholine_pc_colorimetric_assay_kit-e_bc_k796_m) (E-BC-K796-M, Elabscience Biotechnology Co.,Ltd. China) following the manufacturer’s instructions.

Cells (1×10⁶) were seeded into 6-well plates. Intracellular NAD⁺ and NADH in cell extracts were then analyzed. Concentrations were determined using a NAD⁺/NADH Fluorescence Detection Kit (Cat No. S0175, Beyotime), following the manufacturer's instructions strictly.

Cells were seeded at a density of 1×10⁴ cells/well in 96-well plates and processed using the Cell-Based Glucose Uptake Assay Kit (Cat No. S0554S, Beyotime) following the manufacturer's protocol. Absorbance was measured at 450 nm using a microplate reader.

**Measurement of cellular adenosine triphosphate (ATP) and GSH content**

200 µl of lysate was added to each well of a 6-well plate to fully lyse the EAC cells. Centrifugation was performed at 12,000 g for 5 min at 4℃ and the supernatant was removed. The supernatant was used for subsequent assays according to the manufacturer's instructions of the ATP Assay Kit (Cat No. S0026, Beyotime). Briefly, take the appropriate amount of ATP assay reagent and add 100 µl of ATP assay working solution to the assay tube. Allow to stand at room temperature for 3-5 min, add 20 microliters of sample or standard to the assay wells or tubes, mix quickly, and after a minimum interval of 2 s, the RLU values were determined by luminometer.

To determine the level of GSH in EAC cells, the cells were washed once with PBS, collected by centrifugation and the supernatant was aspirated. A 3-fold amount of Protein Removal Reagent M solution was added to the cell sediment volume and fully Vortex. after taking the supernatant, the total glutathione level was determined according to the manufacturer's instructions (Cat No. S0053, Beyotime).

**Glutathione S-transferase (GST) Pull-down**

Glutathione-agarose beads (GE Healthcare Life Sciences, USA) were used to isolate GST fusion proteins. Cells were lysed with Western/IP lysis buffer (Cat No. P0013, Beyotime), placed on ice for 30 min, and then incubated with the beads at 4℃ overnight. The next day, the beads were washed six times with Western/IP lysis buffer and boiled for 10 minutes with 1× Sampling Buffer. The bound proteins were analyzed by western blotting and stained with Caumas Brilliant Blue to analyze protein interactions.

**RNA sequencing and analysis**

RNA sequencing was performed at Glbizzia Biotechnology Ltd (Beijing, China). Briefly, 1×10^6^ EAC cells were harvested and total RNA was extracted using TRIzol reagent (Cat No. AG21102, Accurate Biotechnology, Hunan, China) according to the standard protocol. RNA integrity was assessed using the Fragment Analyzer 5400 (Agilent Technologies, CA, USA). Total RNA was used as the starting material for RNA sample preparation. Sequencing libraries were generated using the NEBNext^®^ UltraTM RNA Library Prep Kit for Illumina^®^ (NEB, USA) according to the manufacturer's recommendations, and index codes were added to assign sequences to each sample. The mRNA was purified from total RNA using poly-T oligo-attached magnetic beads. Fragmentation was performed using divalent cations at elevated temperature in NEBNext First Strand Synthesis Reaction Buffer (5×). First-strand cDNA was synthesised using random hexamer primers and M-MuLV reverse transcriptase (RNase H). Second-strand cDNA was then synthesised using DNA Polymerase I and RNase H. Exonuclease/polymerase activities converted remaining overhangs into blunt ends. After adenylation of the 3' ends of the DNA fragments, NEBNext adaptors with a hairpin loop structure were ligated in preparation for hybridisation.

To preferentially select cDNA fragments of 250~300 bp in length, library fragments were purified using the AMPure XP system (Beckman Coulter, Beverly, USA). Then 3 µl of USER enzyme (NEB, USA) was used with size-selected, adaptor-ligated cDNA at 37℃ for 15 min followed by 5 min at 95℃ before PCR. PCR was then performed using Phusion High-Fidelity DNA Polymerase, Universal PCR primers and Index (X) primers. Finally, PCR products were purified (AMPure XP system) and library quality was assessed using the Qseq 100 instrument. Clustering of the indexed samples was performed on a cBot cluster generation system using the MGIEasy Fast RNA Kit (MGI Tech Co., Ltd., Shenzhen, China) according to the manufacturer's instructions. After cluster generation, the library preparations were sequenced on a DNBSEQ-T7 platform (MGI Tech) and 150 bp paired-end reads were generated.

**Proteomics analysis**

Proteomics analysis was performed at Shanghai Applied Protein Technology Co., Ltd. The extraction of proteins from EAC cells was conducted using a solution of sodium dodecyl sulphate (SDS, 4 %), Tris-HCl buffer (100 mM), DTT (1 mM), and pH 7.6. The quantity of protein was determined using the BCA Protein Assay Kit (Bio-Rad, USA). The protein digestion was conducted in accordance with the Filter-Assisted Sample Preparation (FASP) procedure, as described by Matthias Mann. The peptides resulting from the digestion of each sample were desalted on C18 cartridges (Empore™ SPE Cartridges C18 (standard density), bed I.D. 7 mm, volume 3 ml, Sigma), concentrated by vacuum centrifugation and reconstituted in 40 µL of 0.1 % (v/v) formic acid. Subsequently, 20 µg of protein from each sample was combined with 5 × loading buffer and heated for 5 min. The proteins were subjected to electrophoresis on a 12.5 % SDS-PAGE gel at a constant current of 14 mA for a duration of 90 min. The protein bands were visualised by means of Coomassie Blue R-250 staining.

LC-MS/MS analysis was conducted on a timsTOF Pro mass spectrometer (Bruker), which was coupled to a Nanoelute (Bruker Daltonics) for 60, 120, or 240 min. The peptides were applied to a reverse-phase trapping column (Thermo Scientific Acclaim PepMap100, 100 μm*2 cm, nanoViper C18) connected to the C18 reversed-phase analytical column (Thermo Scientific Easy Column, 10 cm long, 75 μm internal diameter, 3 μm resin) in buffer A (0.1 % formic acid) and separated with a linear gradient of buffer B (84 % acetonitrile and 0.1 % formic acid) at a flow rate of 300 nl/min controlled by IntelliFlow technology. The mass spectrometer was operated in positive ion mode. The mass spectrometer collected ion mobility MS spectra over a mass range of m/z 100 - 1700 and 1/k0 of 0.6 to 1.6, and then performed 10 cycles of PASEF MS/MS with a target intensity of 1.5 k and a threshold of 2500. Active exclusion was enabled with a release time of 0.4 min. Subsequently, the MS raw data for each sample were combined and subjected to identification and quantitation analysis using the MaxQuant 1.5.3.17 software.

**Mass spectrometry**

The reaction solution (1 % SDC/100 mM Tris-HCl, pH=8.5/10 mM TCEP/40 mM CAA) was added to the samples and incubated at 95℃ for 10 min to complete the denaturation, reduction and alkylation of the proteins. The samples were then subjected to centrifugation, after which the supernatants were collected and diluted by the addition of an equal volume of ddH2O. Trypsin was added at a mass ratio of enzyme to protein of 1:50, and the digestion was carried out by incubation and oscillation at 37℃ overnight. The next day, TFA was added to terminate the digestion reaction. The samples were centrifuged at 12,000 g, the supernatants were taken and desalted with a homemade SDB desalting column, vacuum-dried and frozen at -20℃.

Mass spectrometry analyses were performed using a Bruker timsTOF Pro liquid-mass spectrometry system. The UltiMate 3000 RSLCnano system (Thermo) was connected to the timsTOF Pro via a CaptiveSpray nanoliter ion source (Bruker). Pro connection. Peptide samples were bound to a C18 Trap column (75 µm×2 cm, 3 µm particle size, 100 Å pore size, Thermo) via an autosampler feed and separated in a reversed-phase C18 analytical column (75 µm×15 cm, 1.7 µm particle size, 100 Å pore size, IonOpticks). A separation gradient was established by mobile phase A (0.1% formic acid) and mobile phase B (0.1% formic acid/99.9% acetonitrile). The mass spectrometer was operated in PASEF mode with the capillary voltage set to 1500 V. The acquisition range of MS1 and MS2 spectra was set to 100 to 1700 m/z. The ion mobility 1/K0 range was set to 0.75 to 1.4 Vs/cm^2^. The accumulation time and ramp time were set to 100 ms. The acquisition cycle time was 1.16 s and consisted of one full MS1 scan and 10 PASEF MS2 scans. Singly charged parent ions were excluded by the ion mobility signature. Parent ions with signals above 1000 could be singled out for fragmentation. Target intensity was set to 10000.The isolation window of the quadrupole was set to 2.0 Da (m/z 700), 3.0 Da (m/z 800). The collision energy was set to decrease linearly from 59 eV at 1/K0=1.6 Vs/cm^2^ to 20 eV at 1/K0=0.6 Vs/cm^2^ according to the ion mobility setting.The dynamic exclusion time was set to 0.4 min. The mass spectral raw data were analysed using MaxQuant (V2.0.1.0) software, and the database search algorithm used was the built-in Andromeda.

**Biochemical Index Analysis in mouse blood**

Mice were fasted for 12 h, and tail blood was taken to measure fasting blood glucose using a glucometer (Accu-Chek; Roche Diagnostics GmbH, Germany). Mouse INS (insulin) ELISA kit (E-EL-M1382, Elabscience Biotechnology Co.,Ltd. China) was used to determine insulin levels. Fasting insulin resistance index (HOMA-IR) = fasting glucose × fasting insulin/22.5. Next, blood collected from the retro-orbital venous plexus of mice was centrifuged at 2000 r/min for 20 minutes. Plasma was separated and assayed for triglycerides (TG), serum cholesterol (CHO), low-density lipoprotein (LDL), high-density lipoprotein (HDL), tumour necrosis factor-ɑ (TNF-α), interleukin-6 (IL-6), and Interleukin 1 Beta (IL-1β). TG Colorimetric Assay Kit (E-BC-K261-M, Elabscience Biotechnology Co.,Ltd. China), TC Colorimetric Assay Kit (E-BC-K109-M, Elabscience Biotechnology Co.,Ltd. China), LDL Assay Kit (E-BC-K205-M, Elabscience Biotechnology Co.,Ltd. China) and HDL Colorimetric Assay Kit (E-BC-K221-M, Elabscience Biotechnology Co.,Ltd. China) were used to detect TG, CHO, LDL and HDL levels in mouse plasma. Mouse IL-1β ELISA Kit (E-EL-M0037, Elabscience Biotechnology Co.,Ltd. China), mouse IL-6 ELISA kit (E-EL-M0044, Elabscience Biotechnology Co.,Ltd. China) and mouse TNF-α ELISA kit (E-EL-M3063, Elabscience Biotechnology Co.,Ltd. China) were used to detect IL-1β, IL-6 and TNF-α levels.

**Monitoring Cellular Oxygen Consumption Rate (OCR) and Extracellular Acidiﬁcation Rate (ECAR)**

Cellular OCR and ECAR were monitored using the Seahorse XF24 extracellular flux analyser (Seahorse Bioscience, Santa Clara, CA, USA). Briefly, cells (1×10^4^) were seeded onto XF24 cell culture microplates (Seahorse Bioscience) and pre-cultured overnight (37℃, CO_2_-free) in XF base medium (Seahorse Bioscience) containing 1 mM sodium pyruvate, 4 mM glutamax and 11 mM glucose. Basal OCR (ATP-coupled respiration), proton leak from oligomycin (1 µM) treatment and maximal OCR (including residual capacity) from FCCP (1 µM) treatment were monitored. Antimycin A (0.5 µM) was used to confirm mitochondrial OCR. ECAR was monitored and quantified under basal OCR conditions.

**Reference**

1. Page, M.J., et al., *The PRISMA 2020 statement: an updated guideline for reporting systematic reviews.* Bmj, 2021. **372**: p. n71.

**Supplementary figure 1.**

**A**, Flowchart of modeling of nude mice in HFD and ND groups. **B**, Body weight growth curves of mice in HFD and ND groups. Data presented as mean ± SEM with three replicates. *, *p*<0.05; **, *p*<0.01; ***, *p*<0.001. **C**-**E**, Biochemical and inflammatory indices in the blood of mice in the HFD and ND groups. Data presented as mean ± SEM with six replicates. *, *p*<0.05; **, *p*<0.01; ***, *p*<0.001. **F**-**I**, Plasma from mice in the HFD and ND groups was used to intervene with EAC cells, cells were collected for CCK-8 assay (**F**), colony formation assay (**G**), Western blot analysis (**I**), and RT-qPCR analysis (**J**). Data presented as mean ± SEM with three replicates. *, *p*<0.05; **, *p*<0.01; ***, *p*<0.001. **H** and **J**, Heatmap (**H**) and Volcano map (**J**) of the transcriptome sequencing analysis of the transplanted tumors in HFD and ND groups. **L**-**O**, EAC cells were treated with TNF-α, IL-6 and NF-κB inhibitor for 48 hours, cells were collected for RT-qPCR analysis (**L**), Western blot analysis (**M** and **N**), and dual luciferase assay (**O**). Data presented as mean ± SEM with three replicates. ns, not significant; **, *p*<0.01, ***, *p*<0.001.

**Supplementary figure 2.**

**A** and **B**, The prognosis of TRIM15 in EAD (Esophageal adenocarcinoma) (**A**) and the association between TRIM15 and BMI in ESCA (**B**) were determined by the UALCAN-TCGA data platform analysis. *p* values as indicated. **C**, The Barrett's esophagus tissues were analyzed by IHC staining by using the anti-TRIM15 antibody. Red areas, glandular cells with dysplasia; blue areas, glandular cells without dysplasia. D-**F**, OE19 cells were transfected with shTRIM15 plasmids for 72 hours. After puromycin selection, these cells were subcutaneously injected into the nude mice. These mice fed for HFD or ND. Tumor image (**C**), tumor mass (**D**), tumor growth curve (**E**). Data presented as mean ± SEM with six replicates. ***, *p*<0.001.

**Supplementary figure 3.**

**A**-**D**, EAC cells were transfected with EV or HA-TRIM15 plasmids for 48 hours. Cells were collected for Western blot analysis (**A**), RT-qPCR analysis (**B**), CCK-8 assay (**C**), and colony formation assay (**D**). Data presented as mean ± SEM with three replicates. **, *p*<0.01; ***, *p*<0.001. **E**-**I**, The proteomics analysis of OE33 cells after transfection with EV or HA-TRIM15 for 48 hours. Venn diagram (**E**), Domain enrichment analysis (**F** and **G**), Subcellular Localization (**H**), and PPI diagram (**I**). *p* values as indicated.

**Supplementary figure 4.**

**A**, 293T cells were infected with HA-TRIM15 and Flag-YY2 plasmids. After 48 h, Co-IP was performed and the IP samples were analyzed through Western blot analysis. **B** and **C**, EAC cells were transfected with shTRIM15 or shControl plasmids for 72 hours, cells were collected for Western blot analysis (**B**), and RT-qPCR analysis (**C**). Data presented as mean ± SEM with three replicates. ns, not significant. **D** and **E**, EAC or 293T cells were transfected with HA-TRIM15 or EV plasmids for 48 hours, cells were treated with MG132 (10 μM) or CQ (10 μM) for another 24 hours. Then cells were collected for Western blot analysis. **F**-**I**, OE33 cells were transfected with shTRIM15, HA-TRIM15 or HA-∆RING plasmids for 48 hours, cells were treated with Cycloheximide (50 μM) for another 24 hours. Then cells were collected for Western blot analysis at different time points. **J**, TRIM15 protein in OE33 whole cell lysate were pulled down by GST-TRIM15 recombinant proteins. * pointed to the expected molecular weight. **K**-**M**, 293T cells were infected with shTRIM15, HA-TRIM15, HA-∆RING and/or His-Ub (WT, K48, K63) plasmids for 48 hours, cells were treated with MG132 (10 μM) for another 24 hours. Then cells were collected for Co-IP and Western blot analysis.

**Supplementary figure 5.**

**A**-**M**, EAC cells were transfected with shYY2, Flag-YY2, or HA-TRIM15 plasmids for 48 or 72 hours. Cells were collected for Western blot analysis (**A**, **E** and **K**), RT-qPCR analysis (**B** and **F**), CCK-8 assay (**C**, **G** and **L**), colony formation assay (**D**, **H** and **M**), Cellular triglyceride assay (**I**), and Oil Red O Staining (**J**). Data presented as mean ± SEM with three replicates. ns, not significant; **, *p*<0.01; ***, *p<*0.001.

**Supplementary figure 6.**

**A**-**J**, The lipid metabolomics analysis of OE33 cells after transfection with EV or Flag-YY2 for 48 hours. The Principal Component Analysis (PCA) and Partial Least Squares Discriminant Analysis (PLS-DA) (**A**-**D**), Heat map (**E**), lipids count (**F**), differences in lipids content (**G**-**J**). *p* values as indicated.

**Supplementary figure 7.**

**A**, The peak map of the CUT&Tag sequencing analysis after transfection with EV or Flag-YY2 for 48 hours. **B**, Schematic diagram of *FOXRED1* reporter vectors. **C** and **D**, OE33 cells were transfected with Flag-YY2 or shYY2 plasmids for 48 or 72 hours. Cells were harvested for dual luciferase assay. Data presented as mean ± SEM with three replicates. ns, not significant; ***, *p*<0.001.

**Supplementary figure 8.**

**A**-**O**, EAC cells were transfected with shYY2, Flag-YY2, shFOXRED1 or HA-FOXRED1 plasmids for 48 or 72 hours. Cells were collected for Western blot analysis (**A**, **C**, **E**, **I** and **M**), RT-qPCR analysis (**B**, **D**, **F** and **J**), CCK-8 assay (**G**, **K** and **N**) and colony formation assay (**H**, **L** and **O**). Data presented as mean ± SEM with three replicates. ns, not significant; **, *p*<0.01; ***, *p*<0.001.

**Supplementary figure 9.**

**A**-**D**, OE33 cells were transfected with shTRIM15 plasmids for 72 hours. After puromycin selection, these cells were subcutaneously injected into the nude mice. These mice fed for HFD or ND. IHC staining by using the anti-YY2 (**A** and **B**) or anti-FOXRED1 antibody (**C** and **D**). Data presented as mean ± SEM with six replicates. *, *p*<0.05; **, *p*<0.01.

**Supplementary figure 10.**

**A**-**K**, The lipid metabolomics analysis of OE33 cells after transfection with shControl or shFOXRED1 for 72 hours. The PCA and PLS-DA results (**A**-**D**), Heat map (**E**), lipids count (**F**) and differences in lipids content (**G**-**K**). *p* values as indicated.

**Supplementary figure 11.**

**A**-**F**, EAC cells were transfected with shTRIM15, HA-TRIM15, shYY2, or shFOXRED1 plasmids for 48 or 72 hours. Cells were collected for determination of phosphatidylcholine content. Data presented as mean ± SEM with three replicates. ***, *p*<0.001. **G**-**J**, OE33 cells were transfected with shTRIM15, HA-TRIM15FOXRED1 plasmids or/and treated with glycerophospholipids for 72 hours. Cells were collected for CCK-8 assay (**G** and **H**), colony formation assay (**I** and **J**). ns, not significant; ***, *p*<0.001.

**Supplementary figure 12.**

**A**-**X**, EAC cells were transfected with shFOXRED1, HA-FOXRED1, shTRIM15 or HA-TRIM15 plasmids for 48 or 72 hours. Cells and spent medium were collected to measure relative ECAR (**A**, **G**, **M** and **S**), relative OCR (**B**, **H**, **N** and **T**), glucose consumption (**C**, **I**, **O** and **U**), lactate production (**D**, **J**, **P** and **V**), the ECAR (**E**, **K**, **Q** and **W**), and the OCR (**F**, **L**, **R** and **X**). Data presented as mean ± SEM with three replicates. *, *p*<0.05; **, *p*<0.01; ***, *p*<0.001.

**Supplementary figure 13.**

**A**-**B**, EAC cells were transfected with shTRIM15 and/or shFOXRED1 plasmids for 72 hours. Cells were collected to measure ATP content. Data presented as mean ± SEM with three replicates. **, *p*<0.01; ***, *p*<0.001. **C**-**G**, EAC cells were transfected with shTRIM15 or/and shFOXRED1 plasmids for 72 hours. Cells were collected for Western blot analysis (**C** and **D**), CCK-8 assay (**E**), and colony formation assay (**F** and **G**). Data presented as mean ± SEM with three replicates. ns, not significant; **, *p*<0.01; ***, *p*<0.001. **H** and **I**, OE33 cells were transfected with shTRIM15 or/and shFOXRED1 plasmids for 72 hours. After puromycin selection, these cells were subcutaneously injected into the nude mice. The grafted tumor was stained with oil red O. Data presented as mean ± SEM with six replicates. ***, *p*<0.001.

**Supplementary figure 14.**

**A** and **B**, The transcriptome sequencing analysis of OE33 cells after transfection with shFOXRED1 or shControl plasmids for 72 hours. Volcano map (**A**) and KEGG (**B**) enrichment analyses of the RNA-seq data. *p* values as indicated. **C**-**I**, EAC cells were transfected with shFOXRED1, shNDUFS2, shNDUFB9 and/or shControl plasmids for 72 hours, cells were treated with Nicotinamide (2 mM) for another 24 hours. Cells were collected for Western blot analysis (**C**-**E** and **I**), NAD^+^/NADH analysis (**F** and **G**), and Glucose uptake assay (**H**). Data presented as mean ± SEM with three replicates. *, *p*<0.05; **, *p*<0.01. **J**, The ChIP-seq of c-MYC showed the binding peak of c-MYC in the promoter region of *SLC3A2*. **K**, EAC cells were transfected with shc-MYC or shControl plasmids for 72 hours. Cells were collected for RT-qPCR analysis. Data presented as mean ± SEM with three replicates. **, *p*<0.01. **L**-**N**, EAC cells were transfected with shFOXRED1, shc-MYC or shControl plasmids for 48 hours, cells were treated with Everolimus (1 μM) for another 24 hours. Then cells were collected for ChIP-qPCR (**L**) and Western blot analysis (**M** and **N**). Data presented as mean ± SEM with three replicates. ns, not significant; ***, *p*<0.001.

**Supplementary figure 15.**

**A**-**L**, EAC cells were transfected with shYY2, shTRIM15, Flag-SLC3A2, HA-GPX4, shControl or/and EV plasmids for 48 or 72 hours, and treated with RSL3 (1 μM) for another 24 hours. Then cells were collected for intracellular GSH levels assay (**A**, **D**, **H**, and **J**), Western blot analysis (**B**, **G**, and **I**), transmission electron microscopy detection (**C**) (Blue arrows, mitochondria with obvious cristae; Red arrows, shrunken mitochondria) and lipid ROS levels assay (**E** and **F, K** and **L**). Data presented as mean ± SEM with three replicates. *, *p*<0.05; **, *p*<0.01; ***, *p*<0.001.

**Supplementary figure 16.**

**A**-**C**, EAC cells were transfected with shTRIM15 or/and shFOXRED1 plasmids for 48 hours, and treated with IKE (10 μM) for another 24 hours. Then cells were collected for intracellular GSH levels assay. Data presented as mean ± SEM with three replicates. *, *p*<0.05; ***, *p*<0.001. **D**-**H**, EAC cells were transfected with shTRIM15, Flag-YY2, HA-FOXRED1 plasmids for 48 hours, and treated with RSL3 (10 μM) and GPLs for another 24 hours. Then cells were collected for cell viability assay (**D**-**F**), and lipid ROS levels assay (**G** and **H**). Data presented as mean ± SEM with three replicates. **, *p<*0.01; ***, *p*<0.001.
