# Supplementary material for: Obesity‐Associated TRIM15 Promotes the Proliferation of Esophageal Adenocarcinoma Through the YY2/FOXRED1 Axis
Source: Adv Sci (Weinh). 2025 Nov 14;13(4):e17330. doi: 10.1002/advs.202417330 (PMC12822407; doi:10.1002/advs.202417330)
Supplement: Supplementary file 2 — Supporting Information [file ADVS-13-e17330-s004.docx]

**Table S1: The sequences of gene-specific shRNAs**

| **shTRIM15 #1** | 5’-GATCCAGGTGTGAGATGAAGACTTTCTCGAGAAAGTCTTCATCTCACACCTGTTTTTG-3’ |
| --- | --- |
|  | 3’-AATTCAAAAACAGGTGTGAGATGAAGACTTTCTCGAG AAAGTCTTCATCTCACACCTG-5’ |
| **shTRIM15 #2** | 5’-GATCCTTGGCGCATCATCTGGAAATCTCGAGATTTCCAGATGATGCGCCAAGTTTTTG-3’ |
|  | 3’-AATTCAAAAACTTGGCGCATCATCTGGAAATCTCGAGATTTCCAGATGATGCGCCAAG-5’ |
| **shYY2 #1** | 5’-GATCCCAGGCATTGATCTCTCAGATCTCGAGATCTGAGAGATCAATGCCTGGTTTTTG-3’ |
|  | 3’-AATTCAAAAACCAGGCATTGATCTCTCAGATCTCGAGATCTGAGAGATCAATGCCTGG-5’ |
| **shYY2 #2** | 5’-GATCCCTAACGATAACAATGACCAACTCGAGTTGGTCATTGTTATCGTTAGGTTTTTG-3’ |
|  | 3’-AATTCAAAAACCTAACGATAACAATGACCAACTCGAGTTGGTCATTGTTATCGTTAGG-5’ |
| **shFOXRED1 #1** | 5’-GATCGCAGTTCTCATTGCCTGAGAACTCGAGTTCTCAGGCAATGAGAACTGCTTTTTG-3’ |
|  | 3’-AATTCAAAAAGCAGTTCTCATTGCCTGAGAACTCGAGTTCTCAGGCAATGAGAACTGC-5’ |
| **shFOXRED1 #2** | 5’-GATCCCCGCTAGTTGTCAACATGTACTCGAGTACATGTTGACAACTAGCGGGTTTTTG-3’ |
|  | 3’-AATTCAAAAACCCGCTAGTTGTCAACATGTACTCGAGTACATGTTGACAACTAGCGGG-5’ |
| **shc-Myc #1** | 5’-GATCCCTGAGACAGATCAGCAACAACTCGAGTTGTTGCTGATCTGTCTCAGGTTTTTG-3’ |
|  | 3’-AATTCAAAAACCTGAGACAGATCAGCAACAACTCGAGTTGTTGCTGATCTGTCTCAGG-5’ |
| **shc-Myc #2** | 5’-GATCCAGTTGAAACACAAACTTGAACTCGAGTTCAAGTTTGTGTTTCAACTGTTTTTG-3’ |
|  | 3’-AATTCAAAAACAGTTGAAACACAAACTTGAACTCGAGTTCAAGTTTGTGTTTCAACTG-5’ |
| **shNDUFS2 #1** | 5’-GATCCCGAGTGCTGTTTGGAGAAATCTCGAGATTTCTCCAAACAGCACTCGGTTTTTG-3’ |
|  | 3’-AATTCAAAAACCGAGTGCTGTTTGGAGAAATCTCGAGATTTCTCCAAACAGCACTCGG-5’ |
| **shNDUFB9 #1** | 5’-GATCCAGCTGGGAACGAGAGGTTAAGCTCGAGCTTAACCTCTCGTTCCCAGCTTTTTTG-3’ |
|  | 3’-AATTCAAAAAAGCTGGGAACGAGAGGTTAAGCTCGAGCTTAACCTCTCGTTCCCAGCT-5’ |

**Table S2: Antibodies used for western blotting, immunohistochemical staining**

**and ChIP assay.**

| **Antibody** | **Product number** | **Maker** | **Experiment** | **Dilution** |
| --- | --- | --- | --- | --- |
| TRIM15 | 13623-1-AP | Proteintech | Western blotting  Immunohistochemistry  Co-immunoprecipitation | 1/1000  1/300  30 μg/mL cell lysate |
| YY2 | sc-374455 | Santa Cruz Biotechnology | Western blotting  Immunohistochemistry  Chromatin Immunoprecipitation  Co-immunoprecipitation | 1/1000  1/300  30 μg/mL cell lysate  25 μg/mL cell lysate |
| FOXRED1 | 24595-1-AP | Proteintech | Western blotting  Immunohistochemistry | 1/1000  1/300 |
| HA | 51064-2-AP | Proteintech | Western blotting | 1/5000 |
| Flag | 20543-1-AP | Proteintech | Western blotting | 1/20000 |
| pP65 | 82335-1-RR | Proteintech | Western blotting | 1/5000 |
| P65 | 10745-1-AP | Proteintech | Western blotting | 1/5000 |
| SLC3A2 | 15193-1-AP | Proteintech | Western blotting | 1/10000 |
| GPX4 | ab125066 | Abcam | Western blotting | 1/1000 |
| NDUFS2 | 28125-1-AP | Proteintech | Western blotting | 1/1000 |
| NDUFS3 | 15066-1-AP | Proteintech | Western blotting | 1/3000 |
| NDUFV2 | 15301-1-AP | Proteintech | Western blotting | 1/10000 |
| NDUFA9 | 20312-1-AP | Proteintech | Western blotting | 1/2000 |
| NDUFB9 | 15572-1-AP | Proteintech | Western blotting | 1/2000 |
| c-MYC | 10828-1-AP | Proteintech | Western blotting  Chromatin Immunoprecipitation | 1/5000  30 μg/mL cell lysate |
| mTOR | 28273-1-AP | Proteintech | Western blotting | 1/5000 |
| p-mTOR | 67778-1-Ig | Proteintech | Western blotting | 1/5000 |
| β-actin | 20536-1-AP | Proteintech | Western blotting | 1/5000 |
| Ki67 | 27309-1-AP | Proteintech | Immunohistochemistry | 1/3000 |

**Table S3: The sequences of qRT-PCR primers**

| **Gene** | **Forward primer (5′-3′)** | **Reverse primer (5′-3′)** |
| --- | --- | --- |
| *TRIM15* | TCCCTGAAGGTGGTCCATGAG | CAGGATCTTGCCCGAGGATT |
| *YY2* | CACTCCCCTCAGCGTTCTTT | ACCATCAATGGCGGATGGTT |
| *FOXRED1* | ACGGAACATCAATTTTCTTTCAGGA | AAGGACTCCCAAGGACTGGA |
| *SLC3A2* | GTGAAGATCAAGGTGGCGGA | AGGTCGGAGGAGTTAGTCCC |
| *GPX4* | AAGTTCAGTCAGAGACCTGCG | ATATCCGAGCCCTCCTCCTTC |
| *β-actin* | CTCGCCTTTGCCGATCC | GGGGTACTTCAGGGTGAGGA |

**Table S4: The sequences of ChIP-qPCR primers**

| **Gene** | **Forward primer (5′-3′)** | **Reverse primer (5′-3′)** |
| --- | --- | --- |
| *TRIM15* | CCTTCTCCCCGCTACAGTAA | GTCCCCTCTTCTGCTCTCCT |
| *FOXRED1* | GTGAAGAAGTCGAGCATGGC | CCTTGCGATACGTCAGTCCT |
| *SLC3A2* | CTGGTGCCGTGGTCATAATC | GCTCAGGTAATCGAGACGCC |
