# Supplementary figures and images for: Obesity‐Associated TRIM15 Promotes the Proliferation of Esophageal Adenocarcinoma Through the YY2/FOXRED1 Axis
Source: Adv Sci (Weinh). 2025 Nov 14;13(4):e17330. doi: 10.1002/advs.202417330 (PMC12822407; doi:10.1002/advs.202417330)

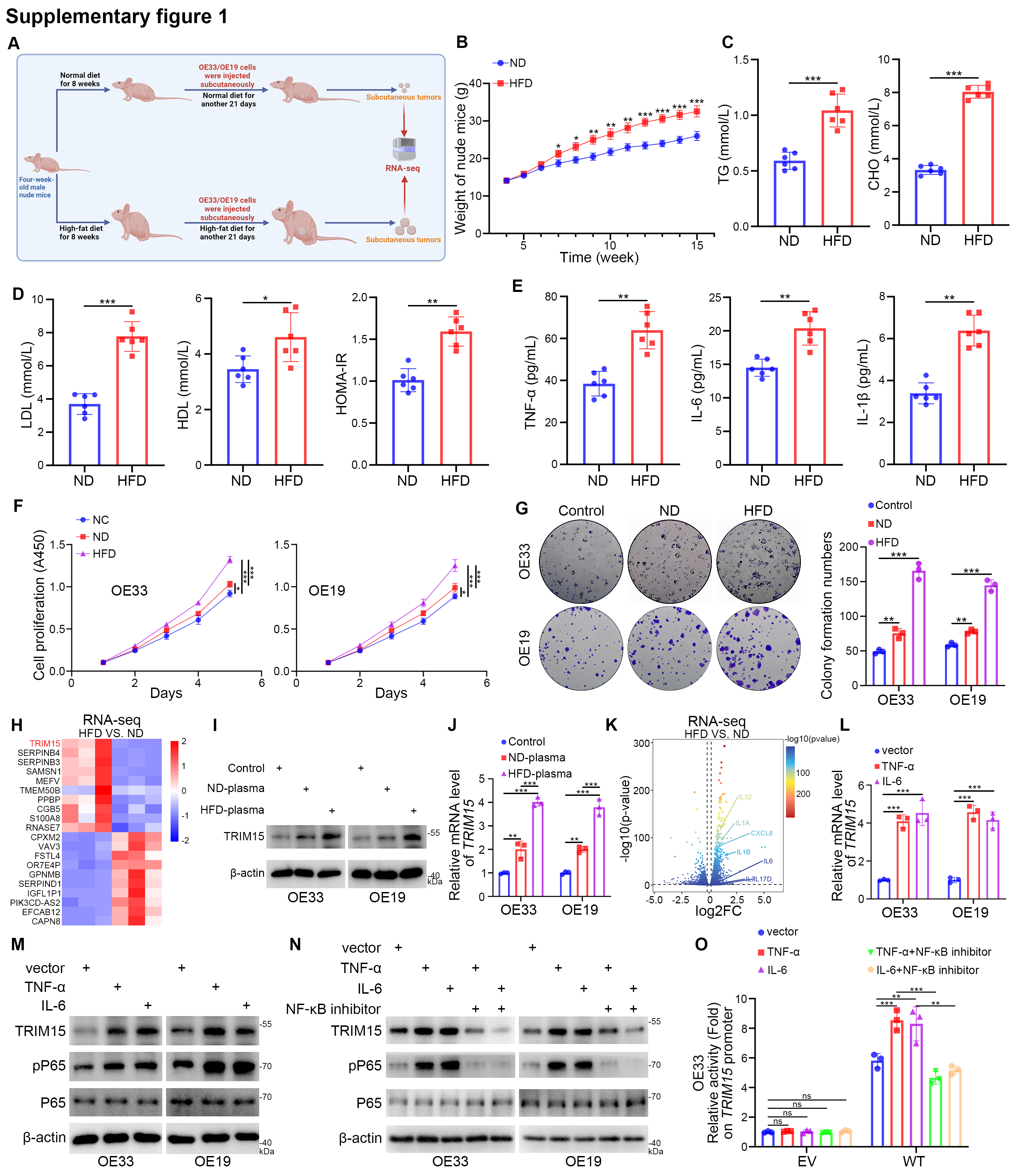

Supplement: Supplementary file 4 — Supporting Information [file ADVS-13-e17330-s002.zip › Supplementary figures/Supplementary figure 1.png]

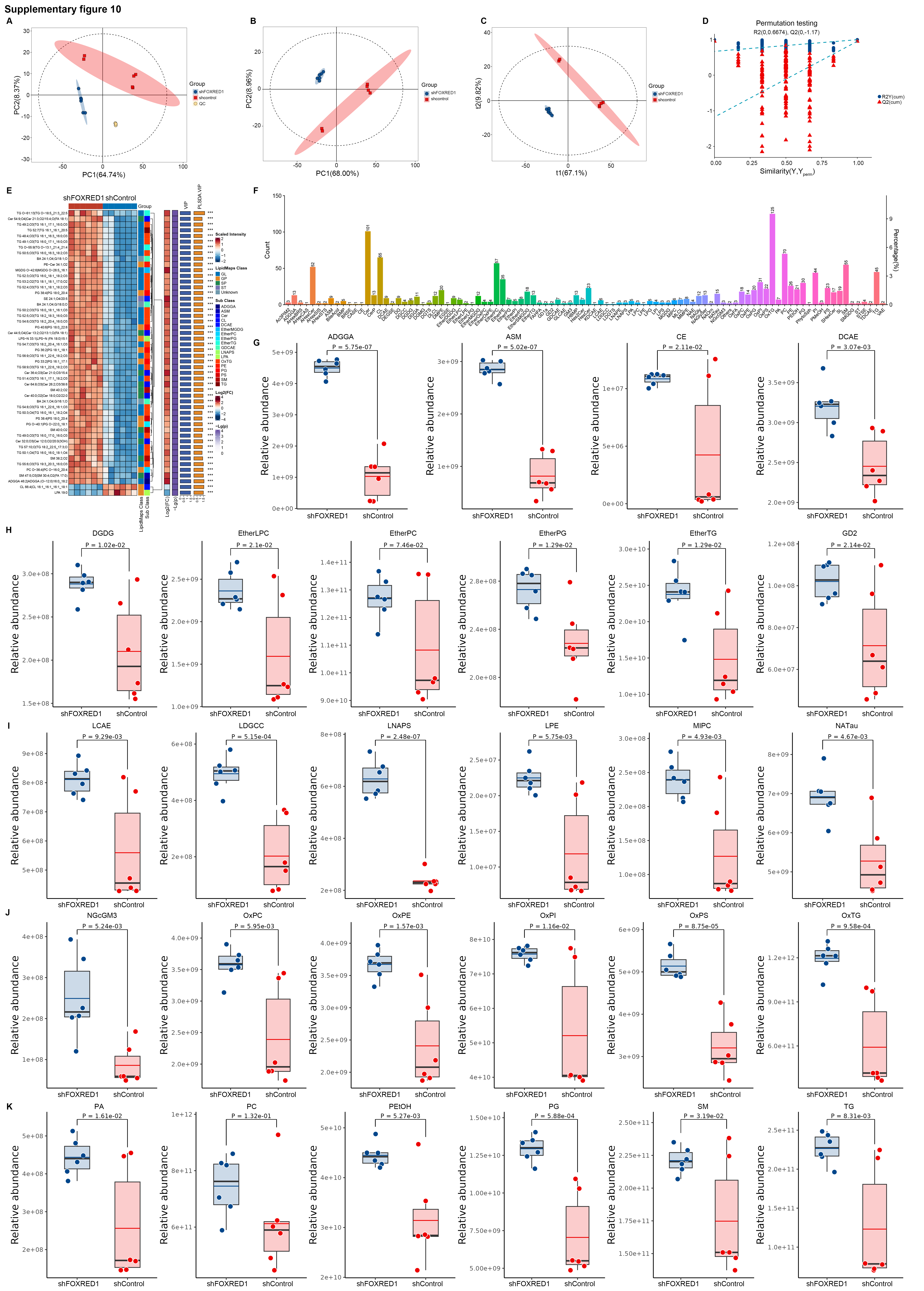

Supplement: Supplementary file 4 — Supporting Information [file ADVS-13-e17330-s002.zip › Supplementary figures/Supplementary figure 10.png]

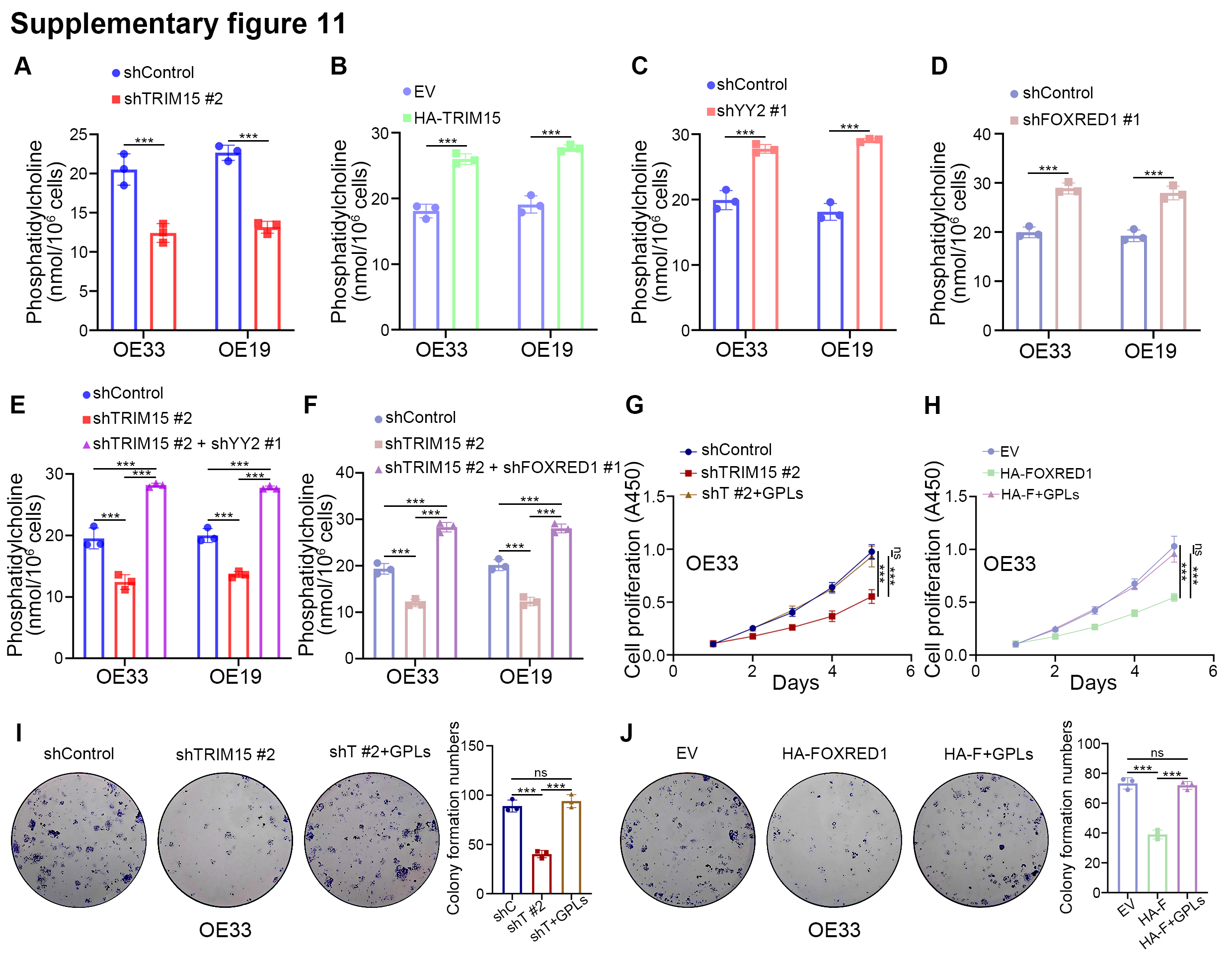

Supplement: Supplementary file 4 — Supporting Information [file ADVS-13-e17330-s002.zip › Supplementary figures/Supplementary figure 11.png]

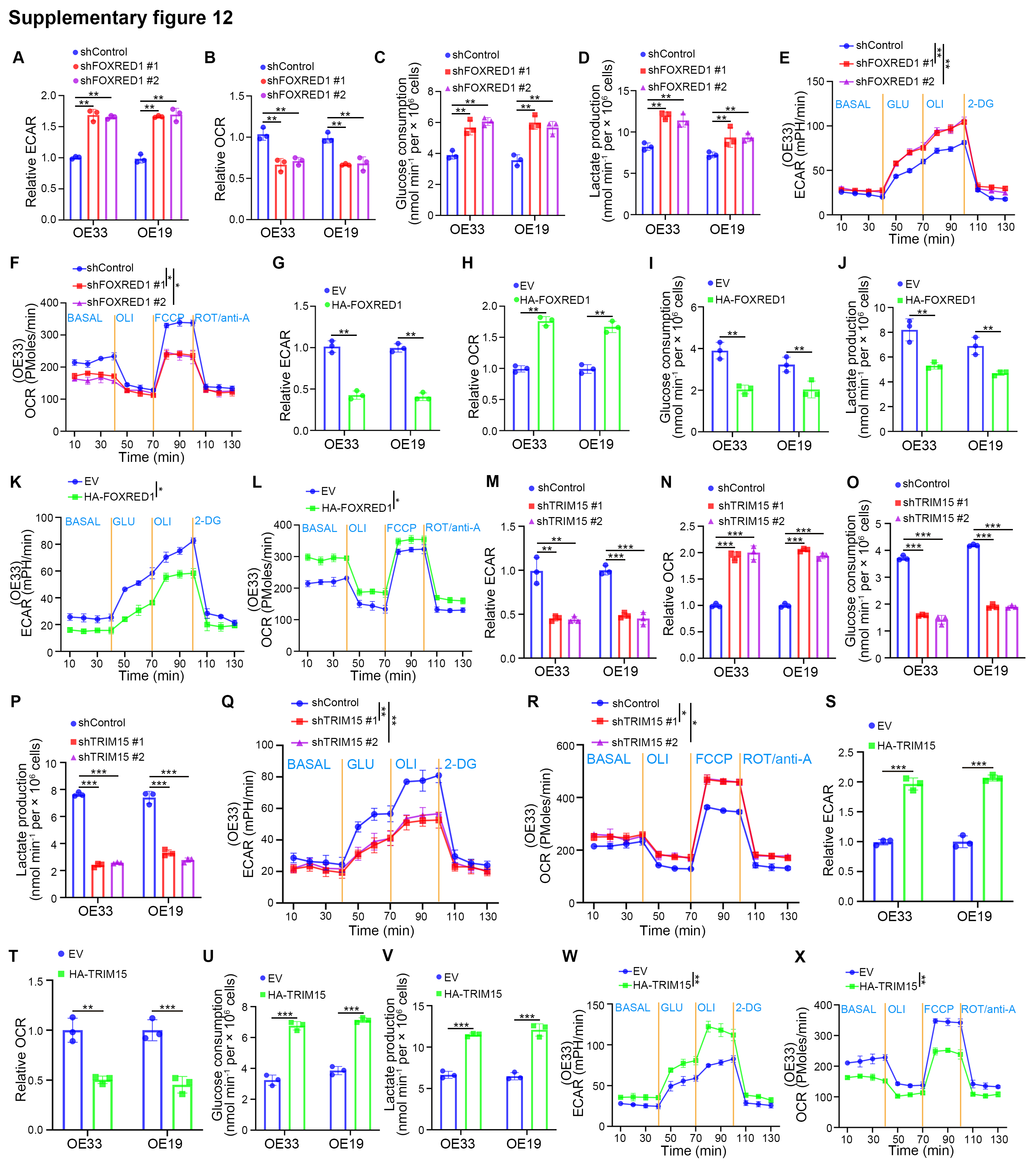

Supplement: Supplementary file 4 — Supporting Information [file ADVS-13-e17330-s002.zip › Supplementary figures/Supplementary figure 12.png]

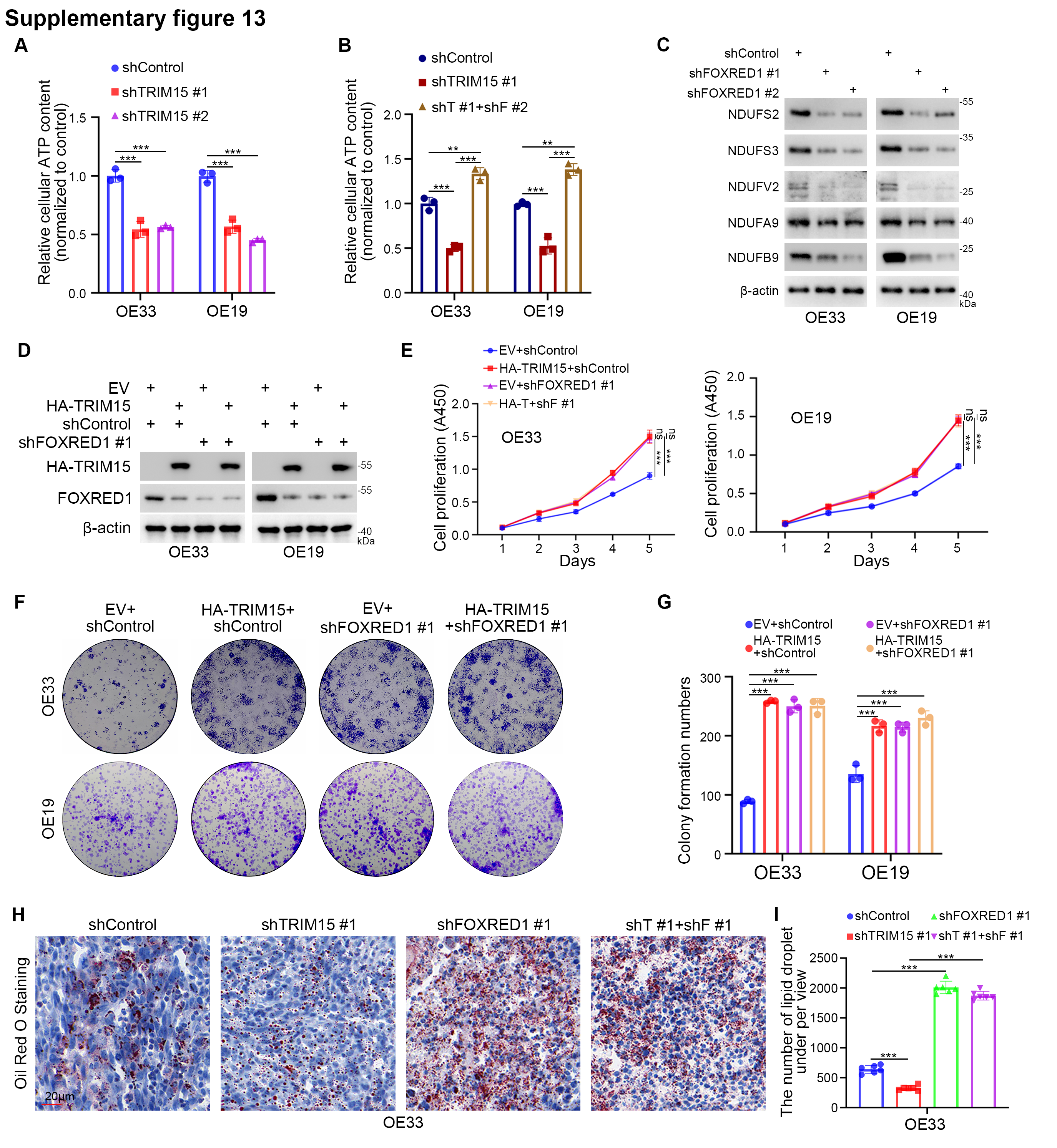

Supplement: Supplementary file 4 — Supporting Information [file ADVS-13-e17330-s002.zip › Supplementary figures/Supplementary figure 13.png]

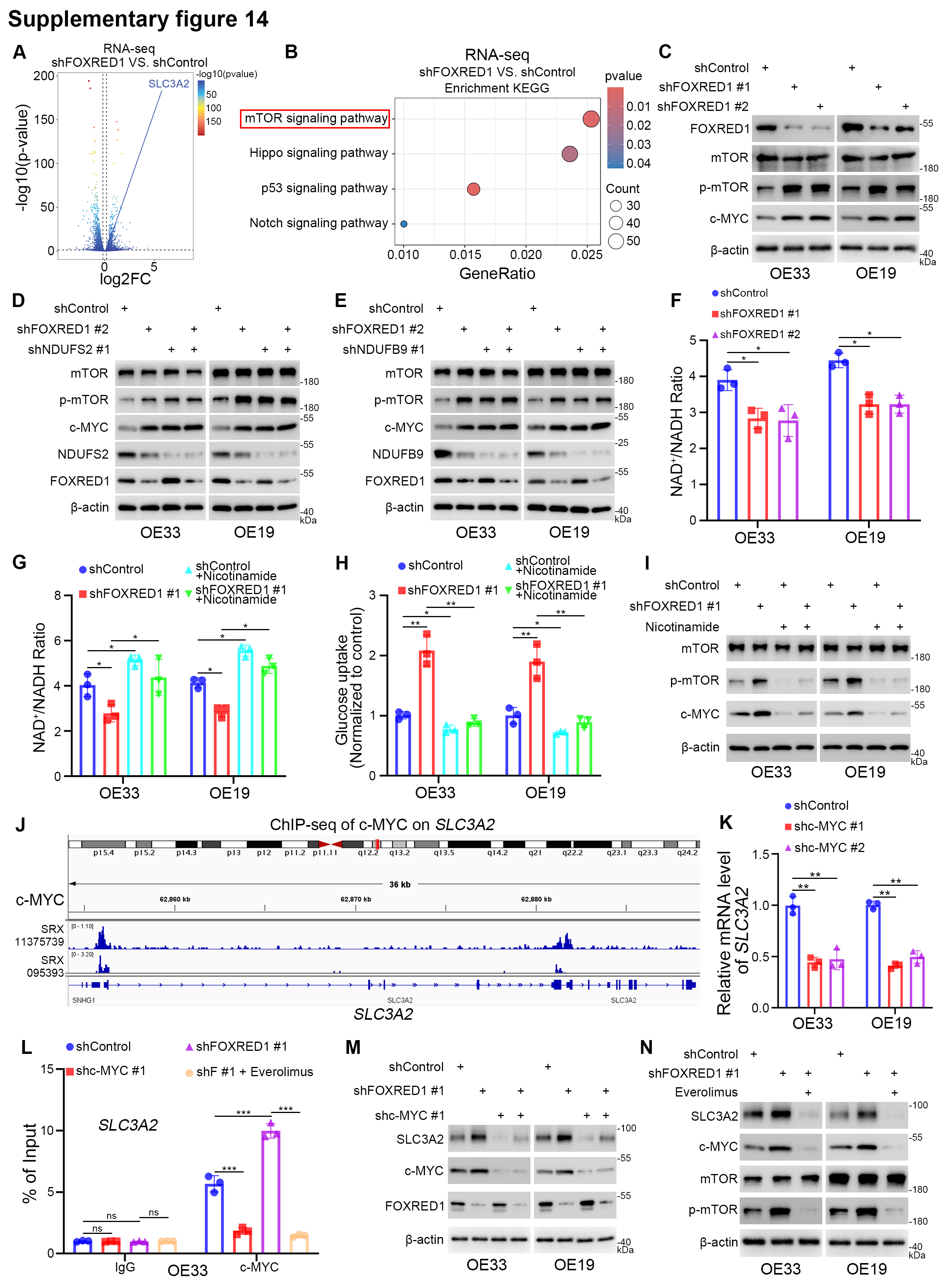

Supplement: Supplementary file 4 — Supporting Information [file ADVS-13-e17330-s002.zip › Supplementary figures/Supplementary figure 14.png]

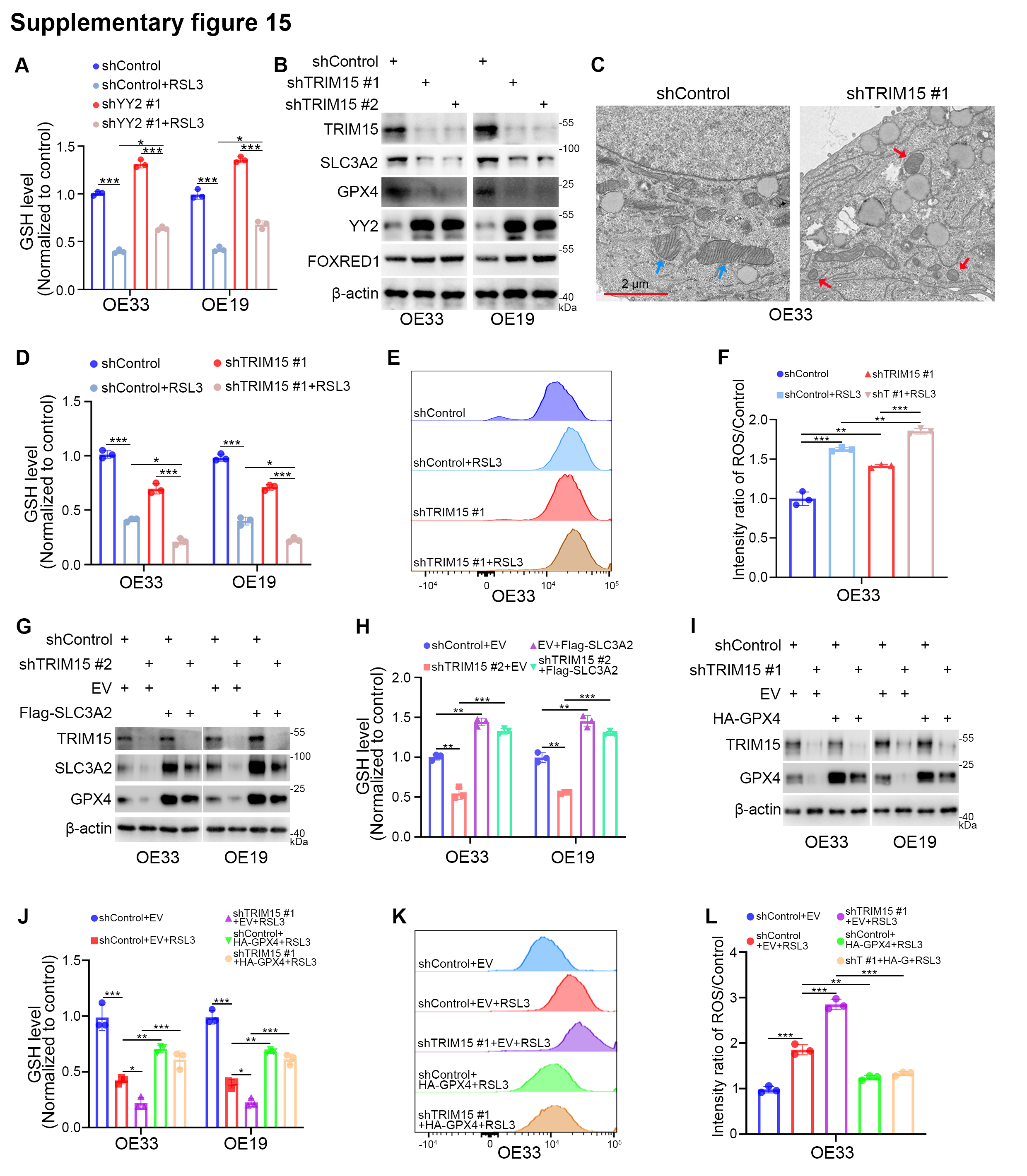

Supplement: Supplementary file 4 — Supporting Information [file ADVS-13-e17330-s002.zip › Supplementary figures/Supplementary figure 15.png]

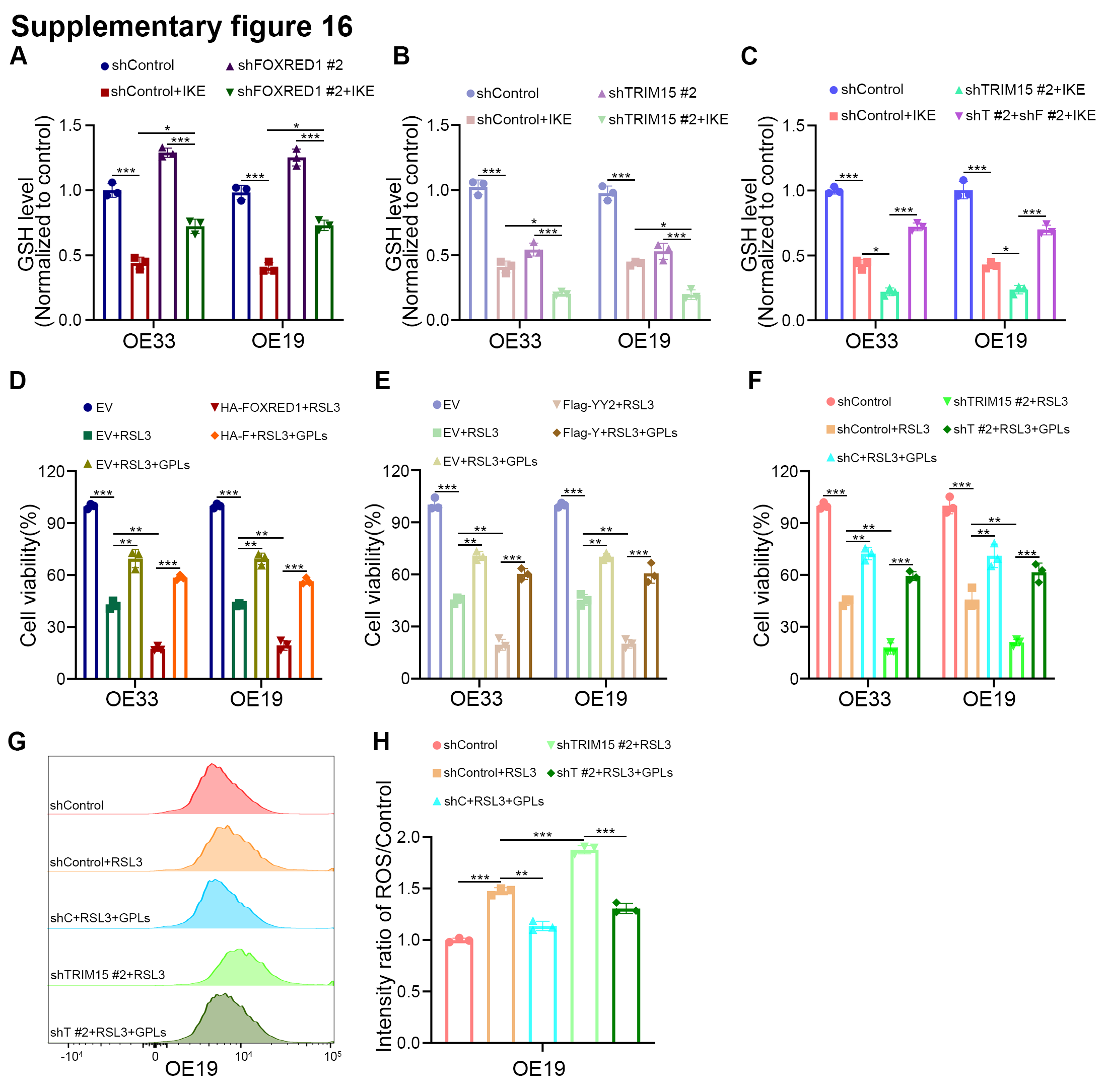

Supplement: Supplementary file 4 — Supporting Information [file ADVS-13-e17330-s002.zip › Supplementary figures/Supplementary figure 16.png]

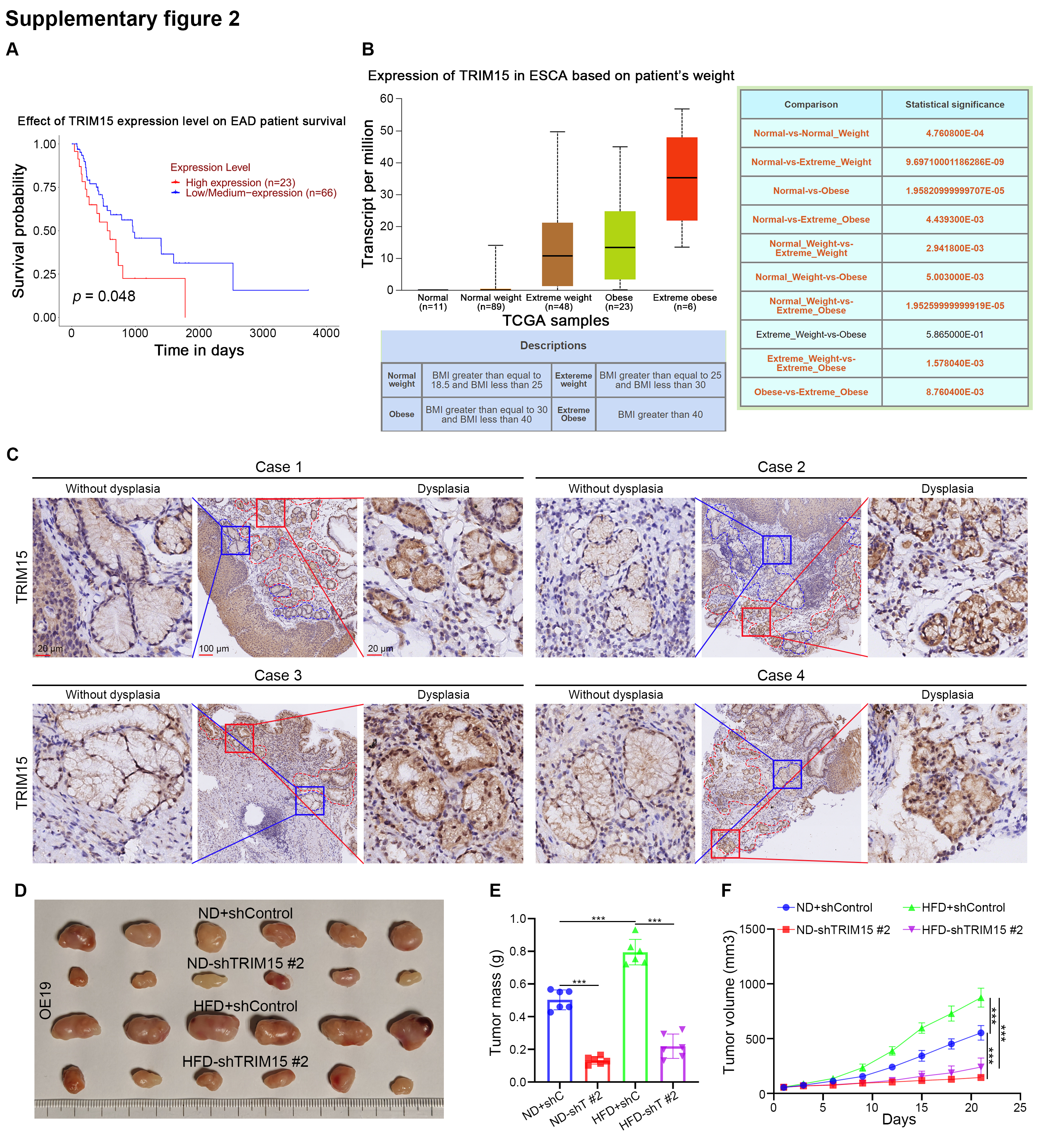

Supplement: Supplementary file 4 — Supporting Information [file ADVS-13-e17330-s002.zip › Supplementary figures/Supplementary figure 2.png]

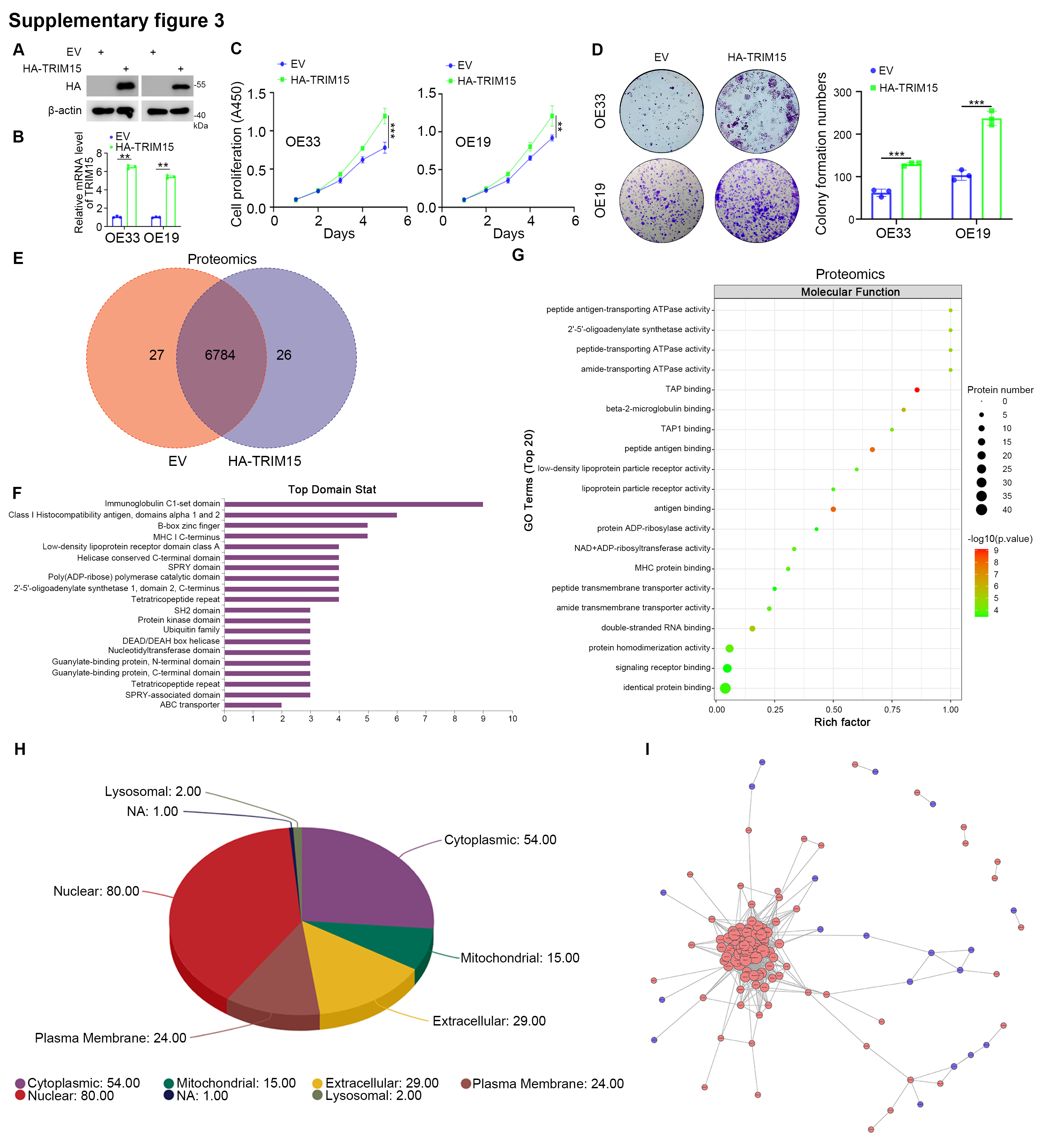

Supplement: Supplementary file 4 — Supporting Information [file ADVS-13-e17330-s002.zip › Supplementary figures/Supplementary figure 3.png]

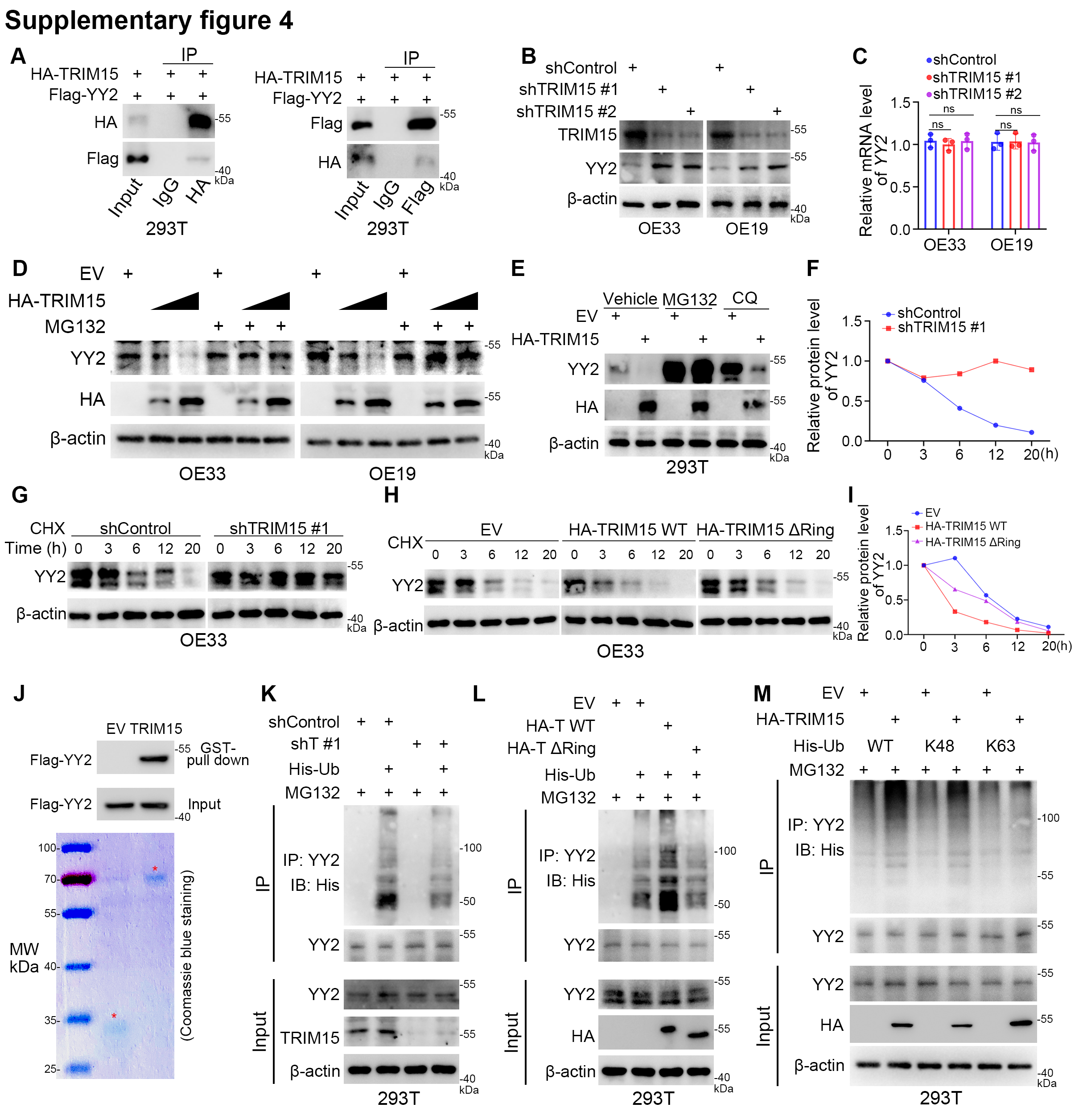

Supplement: Supplementary file 4 — Supporting Information [file ADVS-13-e17330-s002.zip › Supplementary figures/Supplementary figure 4.png]

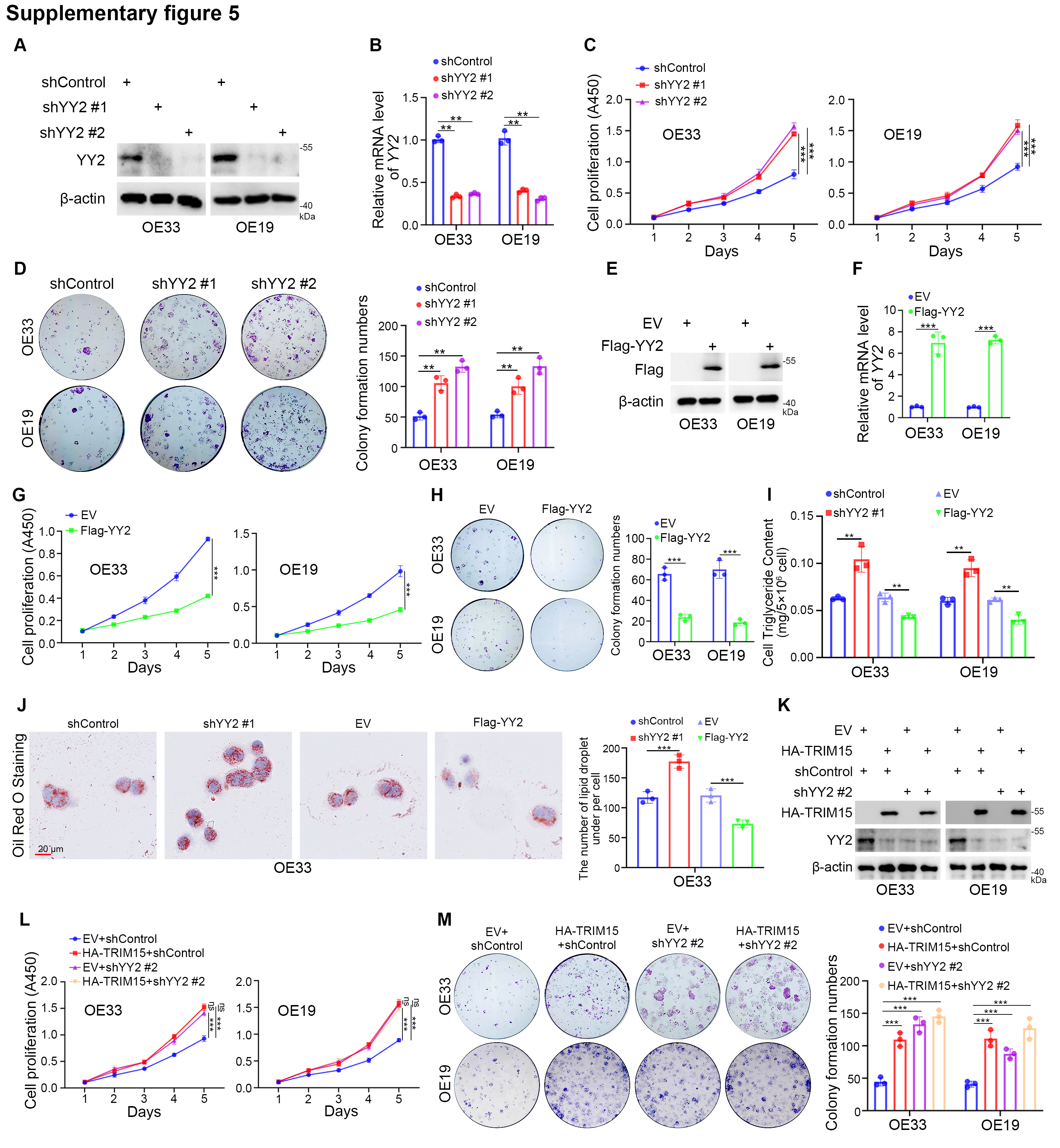

Supplement: Supplementary file 4 — Supporting Information [file ADVS-13-e17330-s002.zip › Supplementary figures/Supplementary figure 5.png]

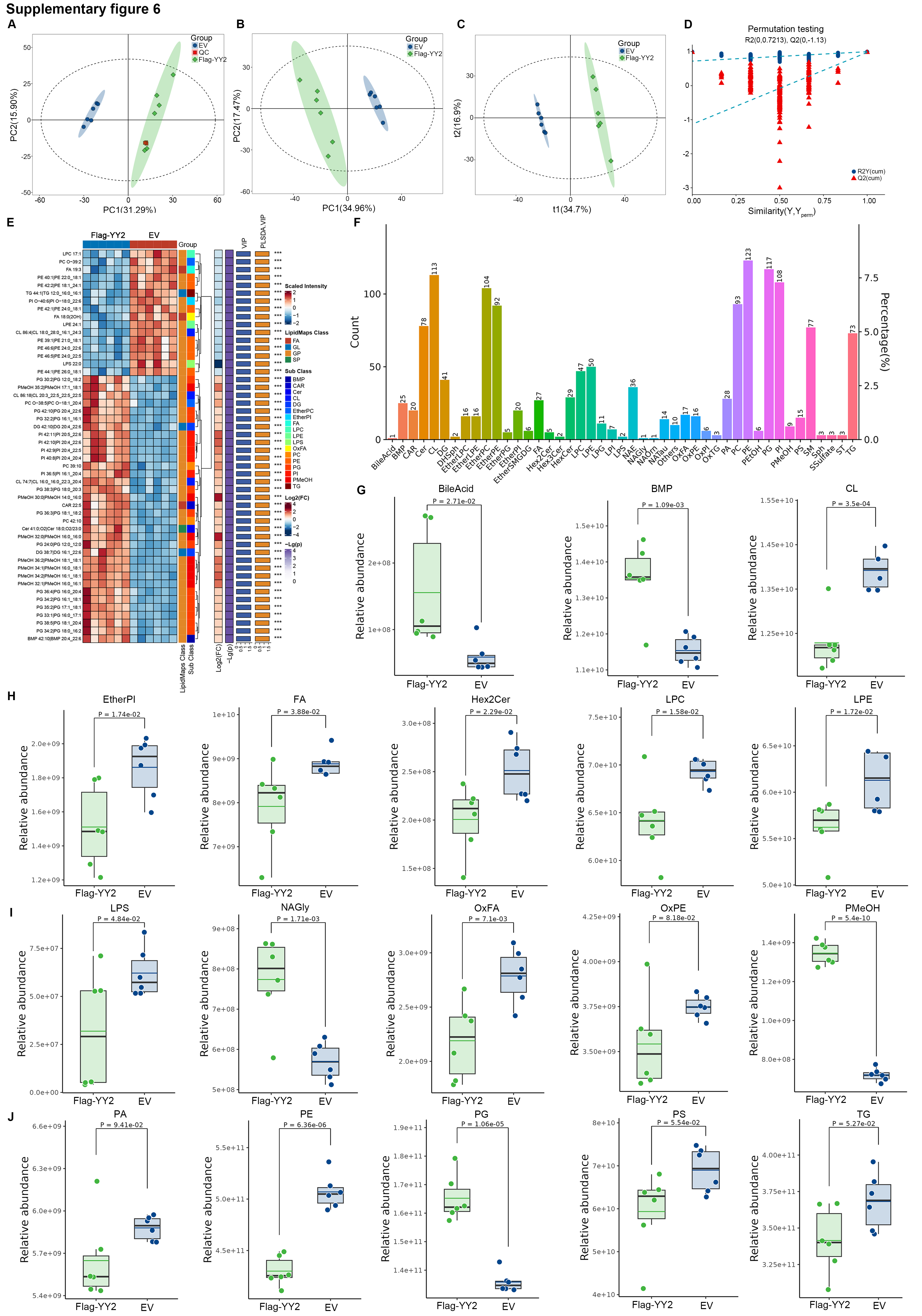

Supplement: Supplementary file 4 — Supporting Information [file ADVS-13-e17330-s002.zip › Supplementary figures/Supplementary figure 6.png]

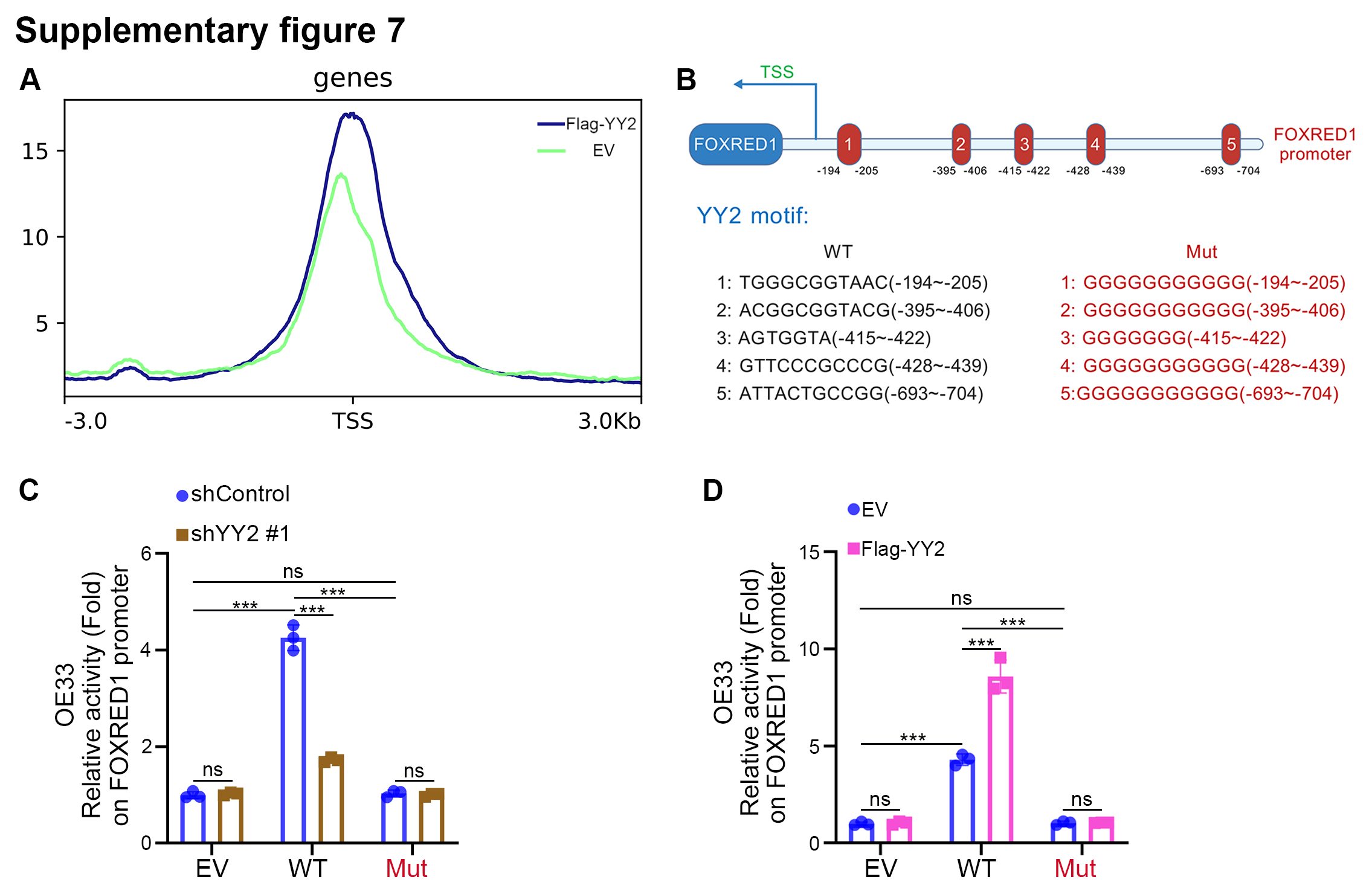

Supplement: Supplementary file 4 — Supporting Information [file ADVS-13-e17330-s002.zip › Supplementary figures/Supplementary figure 7.png]

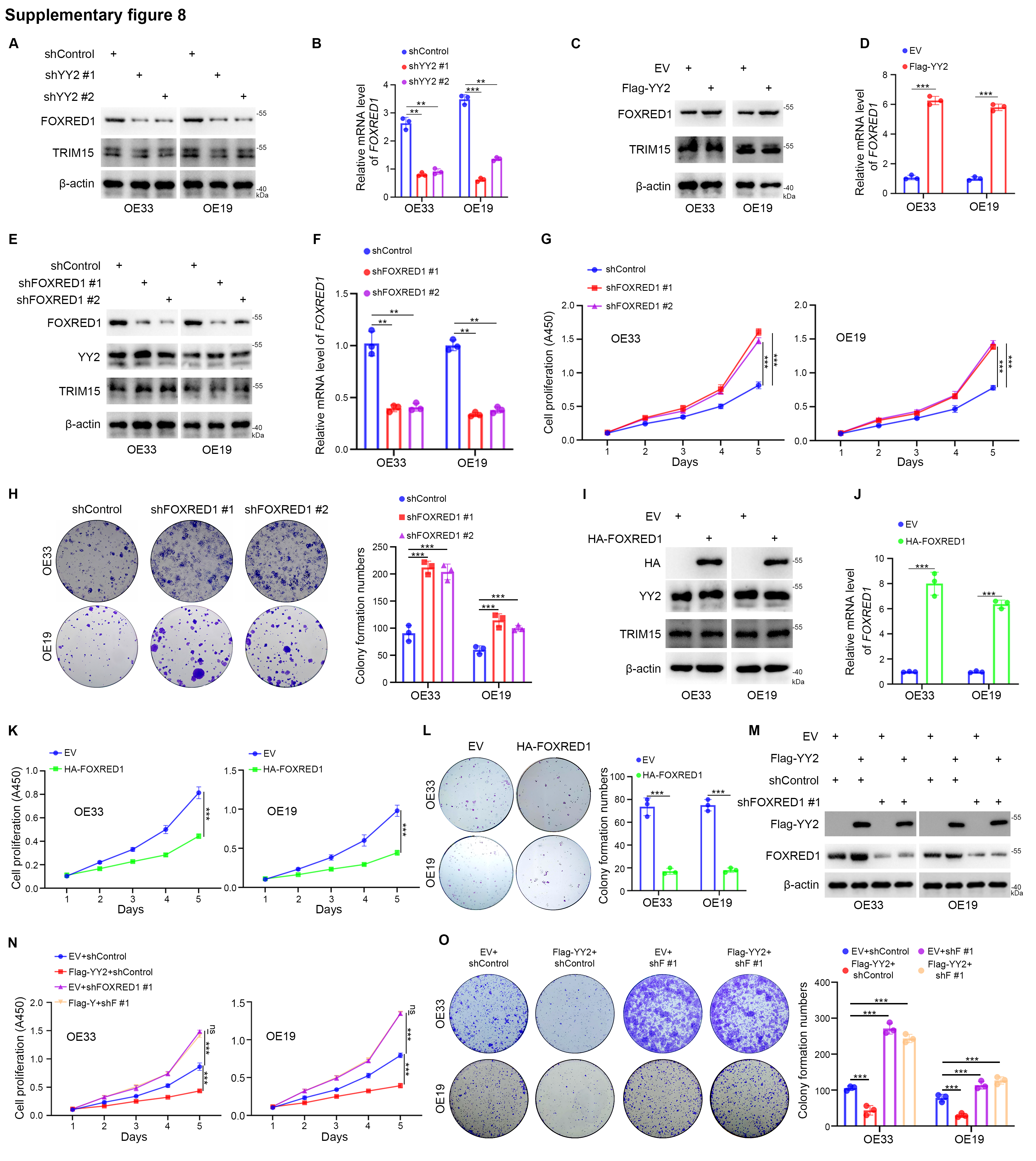

Supplement: Supplementary file 4 — Supporting Information [file ADVS-13-e17330-s002.zip › Supplementary figures/Supplementary figure 8.png]

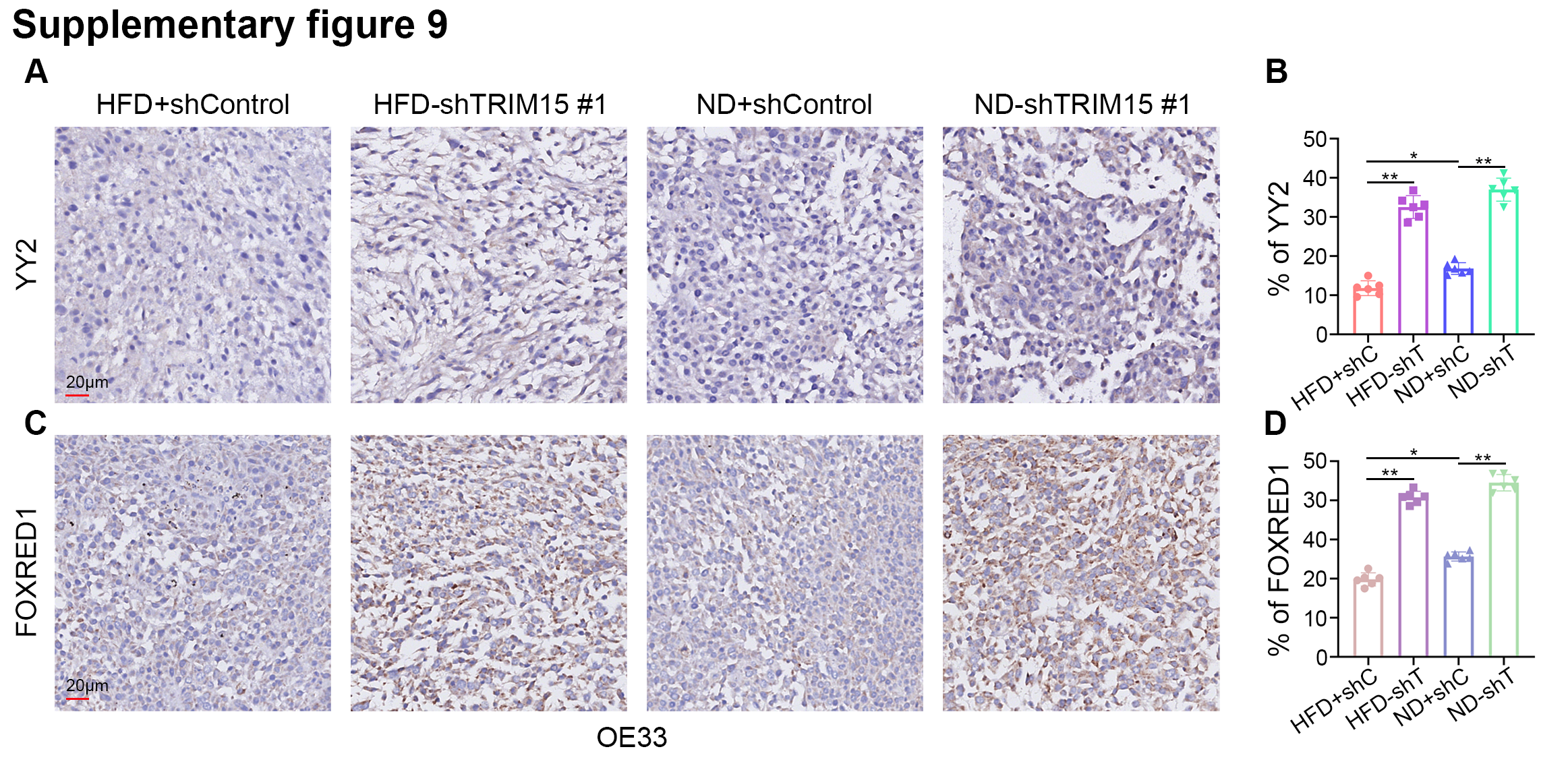

Supplement: Supplementary file 4 — Supporting Information [file ADVS-13-e17330-s002.zip › Supplementary figures/Supplementary figure 9.png]
